# Speciation of Oxygen Functional Groups on the Carbon Support Controls the Electrocatalytic Activity of Cobalt Oxide Nanoparticles in the Oxygen Evolution Reaction

**DOI:** 10.1021/acsami.2c18403

**Published:** 2023-01-19

**Authors:** Aleksander Ejsmont, Karolina Kadela, Gabriela Grzybek, Termeh Darvishzad, Grzegorz Słowik, Magdalena Lofek, Joanna Goscianska, Andrzej Kotarba, Paweł Stelmachowski

**Affiliations:** † Department of Chemical Technology, Faculty of Chemistry, Adam Mickiewicz University in Poznań, Uniwersytetu Poznańskiego 8, 61-614Poznań, Poland; ‡ Faculty of Chemistry, Jagiellonian University, Gronostajowa 2, 30-387Krakow, Poland; § Department of Chemical Technology, Faculty of Chemistry, Maria Curie-Sklodowska University in Lublin, Maria Curie-Sklodowska Sq. 3, 20-031Lublin, Poland

**Keywords:** hydrogen production, mesoporous carbon, nanoparticles, surface modification, plasma oxidation, oxygen functional groups

## Abstract

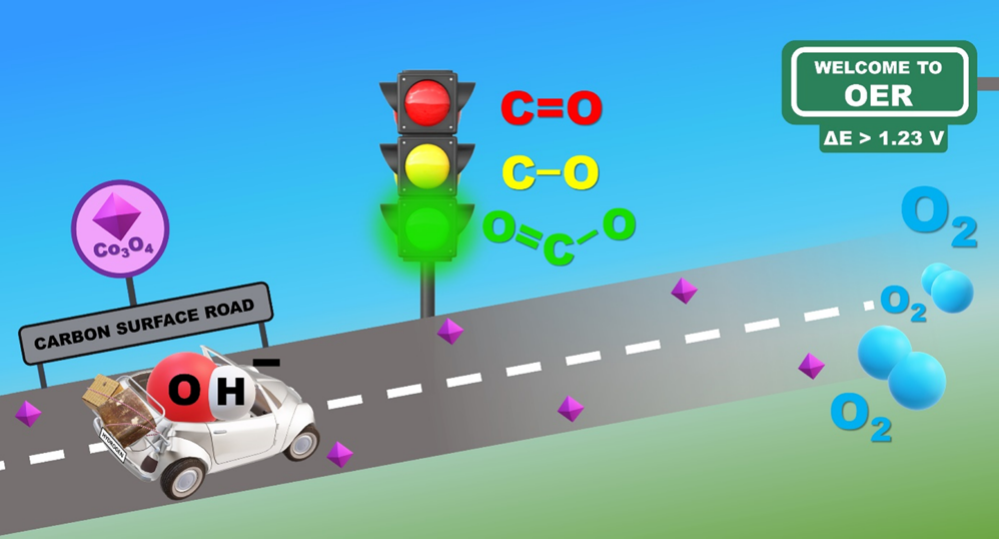

The effective use of the active phase is the main goal of the optimization of supported catalysts. However, carbon supports do not interact strongly with metal oxides, thus, oxidative treatment is often used to enhance the number of anchoring sites for deposited particles. In this study, we set out to investigate whether the oxidation pretreatment of mesoporous carbon allows the depositing of a higher loading and a more dispersed cobalt active phase. We used graphitic ordered mesoporous carbon obtained by a hard-template method as active phase support. To obtain different surface concentrations and speciation of oxygen functional groups, we used a low-temperature oxygen plasma. The main methods used to characterize the studied materials were X-ray photoelectron spectroscopy, transmission electron microscopy, and electrocatalytic tests in the oxygen evolution reaction. We have found that the oxidative pretreatment of mesoporous carbon influences the speciation of the deposited cobalt oxide phase. Moreover, the activity of the electrocatalysts in oxygen evolution is positively correlated with the relative content of the COO-type groups and negatively correlated with the C=O-type groups on the carbon support. Furthermore, the high relative content of COO-type groups on the carbon support is correlated with the presence of well-dispersed Co_3_O_4_ nanoparticles. The results obtained indicate that to achieve a better dispersed and thus more catalytically active material, it is more important to control the speciation of the oxygen functional groups rather than to maximize their total concentration.

## 1 Introduction

Carbon-based materials are extensively studied in the context of their electrocatalytic applications.^1^ The research with carbon materials is so widespread because of their relatively high electrical conductivity, the possibility of obtaining materials with very high specific surface areas (SSAs), and their susceptibility to various surface modifications.^2−6^ To enhance the deposition of the catalytically active phases such as metal oxides, sulfides, phosphides, and so forth, carbon materials are often subjected to electrochemical oxidative pretreatment^7,8^ or that with concentrated acids.^9,10^ However, plasma oxidation has been used more and more extensively to modify the surface of carbon materials.^11−13^ It has been shown that optimized surface oxidation of carbon materials leads to an enhanced adsorption capacity of transition metals.

To date, the mechanisms of adsorption of metal cations on surface oxygen groups of carbon materials have not been explicitly explained. Various factors such as morphology and degree of graphitization of carbon, pH, concentration of cations, and type of anions influence the removal of cations from aqueous solutions.^14^ Heavy-metal ions, such as Cu^2+^, are mainly bonded by complexation of the surface carboxylic groups,^15,16^ while Cd^2+^ adsorption occurs mainly on carbonyl groups and aromatic structures, where dipole–dipole interactions (as cation−π bonding) are preferred.^17^ Adsorption of Co^2+^ and Ni^2+^ was found to occur primarily on carboxylic surface groups,^18^ and electrostatic interactions between cations and negatively charged oxygen groups were proposed.^15^ Considering the binding of transition-metal oxides with carbon functional groups, it has been shown that CuO nanoparticles (49 nm in diameter) interact with hydroxyl groups,^19^ while TiO_2_ nanoparticles (20 nm in diameter) interact also with carbonyl groups^20^ through physical interactions or even chemical bonding, including hydrogen bond^21^ and coordinate bond.^22^ Strong interactions between metal oxides and modified carbons allow for obtaining material with stable nanoparticles.^23^ The surface properties of the carbon supports are also crucial for the stability of the deposited metallic nanoparticles. Cobalt nanoparticles have been shown to be more dispersed on functionalized carbon due to their interactions with carbon and oxygen atoms, resulting in tight anchoring of cobalt on the substrate and also preventing aggregation.^24^

Cobalt in its metallic or oxide forms constitutes an active phase for many catalytic processes. In particular, for energy-related electrochemical applications, it is one of the most researched elements.^25−27^ Among the wide range of cobalt-based catalysts for oxygen evolution reaction (OER), some stand out due to their remarkable properties, such as ultra-low overpotential, for example, FeCoW (191 mV at 10 mA cm^–2^),^28^ self-healing ability (CoP_i_ and CoB_i_),^29^ or outstanding long-term stability, for example, CoFe_2_O_4_ of 4000 cycles.^30^ A large diversity of materials hampers comparative analysis, as often disparate parameters are reported, and rarely is the full set of requirements met, such as high access to multiple active centers, initial water adsorption ability, and low cost and facile preparation. Until now, Co_3_O_4_ spinel has been one of the most extensively studied catalysts for oxygen generation and is being used alone or as an additive to other materials to improve their performance. Two specific cobalt sites are present in Co_3_O_4_ spinel: tetrahedral sites coordinated by four oxygen atoms and octahedral sites coordinated by six oxygen atoms. The octahedral Co^3+^ and adjacent oxygen atoms are located in the Co_4_O_4_ cubane configuration, which has been implied to be essential for OER.^31^ However, there are reports specifying that Co^2+^ enables the formation of a cobalt oxyhydroxide intermediate (CoOOH), which is the main active species for oxygen generation, while Co^3+^ and its transformation to Co^2+^ ensure the formation of oxygen vacancies to maintain charge balance.^32^ Despite the OER activity of cobalt oxides alone, the introduction of other metals into the crystal lattice is a typical technique to increase their performance. Zhang et al.^33^ after introducing 13 different metals into ultrathin Co_3_O_4_ nanosheets showed that Fe-Co_3_O_4_ exhibits the highest stability for 50 h of the process and an overpotential of 262 mV (at 10 mA cm^–2^). Heteroatom enrichment of Co_3_O_4_ is also proposed to increase the number of active sites, for example, induction by lithium addition^34^ or leaching/activation mechanism by substitution with magnesium.^35^ However, multifunctional heteroatom catalysts often corrode, especially in an acidic environment, hence they have a limited scope of implementation.^36^ A different approach is the formation of composites, for instance, with metal–organic frameworks (MOFs).^37^ A comparison of Co_3_O_4_ alone with the Co_3_O_4_@MOF-74 core–shell structure showed that at the same current density (50 mA cm^–2^), the composite required lower overpotential (285 mV) than pristine metal oxide (336 mV). However, the various cobalt oxide systems display low conductivity, deficient active centers, and low water adsorption, ranking them below highly efficient IrO_2_-based catalysts.^33^ An interesting approach to consider is the application of porous carbons as support for Co_3_O_4_. Porous carbons have large SSAs and graphitic domains in the structure, as well as are prone to surface functionalization. For instance, Lei et al. proposed Co_3_O_4_ with N-doped CMK-3 carbon as a composite, which indicated good stability for 1000 cycles and comparable activity in OER as noble Pt/C + IrO_2_ catalysts.^38^ Comparing the potential values for half-wave oxygen reduction reaction (ORR) and OER, it was shown that Δ*E* is 0.762 V for the Co_3_O_4_-carbon composite and 0.741 V for Pt/C + IrO_2_. Furthermore, the slope of the Tafel plot for carbon-supported Co_3_O_4_ is 81 mV dec^–1^, which proves its high efficiency and cost-effectiveness. A similar example is Co/Co_3_O_4_ prepared in situ in a hierarchically porous carbon structure described by Zhang et al.^39^ The group showed that their metal/metal oxide/porous carbon system has the same or higher OER activity than commercial Pt/C or Ir/C. Therefore, it could be stated that making attempts at understanding the activity of cobalt oxide-carbon composites is of great importance and can contribute to creating more efficient OER catalysts.

Despite the large abundance of studies on carbon-based composites in the OER, reports on the effect of oxygen group speciation on the carbon surface electronic properties, adsorptive properties and catalytic activity of carbon-supported catalysts,^24,40^ and especially electrocatalysts are scarce. One suggestion is the participation of the carbon support by means of the oxygen spillover mechanism.^41^ In contrast, for the ORR, surface functional groups were found to play a key role in the dispersion of the active phase and influence the electron-transfer pathway,^42−44^ while the metal oxide–carbon heterojunction was shown to promote synergistic ORR with a cooperated two-step electrocatalysis.^45^

In this study, our objective was to evaluate the role of different surface oxygen groups on carbon support on the quantity and dispersion of the cobalt oxide, Co_3_O_4_, as a model but also as a practical active phase.^46^ Furthermore, the goal was to establish whether the speciation of surface oxygen groups, determined with X-ray photoelectron spectroscopy (XPS), plays a role in the OER. We used oxygen plasma treatment, which provides an effective and fast means of preparing carbons with different speciation of oxygen functional groups, without deteriorating the basic physicochemical properties of the carbons. To the best of our knowledge, such an analysis has not been performed to date, and the results obtained provide a novel strategy for the development of carbon-based electrocatalysts with possible applications in the field of electrochemical energy conversion and storage.

## 2 Materials and Methods

### 2.1 Materials

To synthesize graphitic ordered mesoporous carbon with a cubic structure, the nanocasting technique was utilized. In the first stage, the ordered mesoporous silica KIT-6 was obtained, which was used as a solid matrix for carbon. Subsequently, carbon material C_KIT-6_ was prepared, followed by its chemical oxidation to improve carbon surface chemistry.

#### Preparation of KIT-6 via Hydrothermal Method

In a bottle made of high-density polyethylene, 4 g of the triblock copolymer Pluronic P123 (EO_20_PO_70_EO_20_, Aldrich, St. Louis, MO, USA) was dissolved in a hydrochloric acid solution (144 g of distilled water and 7.9 g of hydrochloric acid, Avantor Performance Materials Poland S.A.) at 35 °C. To the continuously mixed solution, 4 g of butyl alcohol (Polskie Odczynniki Chemiczne) was poured in, followed by 8.6 g of tetraethyl orthosilicate (98 wt %, Aldrich) addition in a dropwise manner. The mixture was stirred vigorously for 24 h at 35 °C. Subsequently, the solution was sealed in the bottle, placed in the oven, and subjected to hydrothermal treatment for the next 24 h at 100 °C. The resulting white precipitate was vacuum filtered, washed three times with distilled water, and left for drying for 12 h at 100 °C. Last, the material was calcined for 8 h at 550 °C in air, wherein the copolymer decomposed.

#### Preparation of C_KIT-6_ via Nanocasting

The as-prepared KIT-6 silica template was impregnated twice with a sucrose acidic solution. To the solution consisting of 5 mL of water and 0.14 mL of sulfuric(VI) acid (Avantor Performance Materials Poland S.A., Gleiwitz, Poland), 1.25 g of sucrose (Aldrich) was added. 1.0 g of fine silica powder was then uniformly treated with a sucrose solution, followed by heating for 6 h at 100 °C and then for 6 h at 160 °C. The silica–carbon composite was again impregnated with a less concentrated sucrose solution, containing 0.8 g of sucrose, 0.09 mL of sulfuric(VI) acid, and 5 mL of distilled water. The material was then heated again in the same two-stage manner. The efficiently impregnated template with carbon source was next pyrolyzed for 3 h at 900 °C in an argon atmosphere, with a heating rate of 2.5 °C min^–1^. After the process, the residue of silica was washed out twice by using 200 mL of 5% hydrofluoric acid solution (Avantor Performance Materials Poland S.A.). Finally, the carbon material was washed with distilled water and ethanol three times, followed by drying for 12 h at 100 °C. The pristine carbon material was labeled as C_KIT-6_-ref.

#### Oxidation of C_KIT-6_ with Ammonium Persulfate

The C_KIT-6_ has been subjected to the chemical oxidation process, using an acidic ammonium persulfate (APS, Aldrich) solution. In a round-bottomed flask, 1 g of a fine powder of C_KIT-6_ was flooded with 60 mL of 1 mol l^–1^ APS solution. The suspension was vigorously stirred and heated under reflux for 6 h at 60 °C. The material was then vacuum filtered, washed three times with distilled water and ethanol, and in the end dried for 12 h at 100 °C. The oxidized carbon material was denoted as C_KIT-6_-APS.

#### Oxidation of C_KIT-6_ with Plasma

To modify C_KIT-6_ powders, a commercial cold plasma system with a generator frequency of 40 kHz was used (Femto-Diener Electronic GmbH, Nagold, Germany). Pure oxygen was used as a feed gas for plasma generation (Air Products, 99.9998% O_2_). The variable parameters were the time of plasma treatment, power of the generator, and pressure inside the plasma chamber. For preliminary characterization of plasma-oxidized C_KIT-6_, we used 1 and 10 min of modification time with 0.2 mbar of oxygen pressure and 100 W of generator power. To obtain a spectrum of carbons with surfaces functionalized with different oxygen groups, we used plasma modification with parameters summarized in Table 1. Plasma-modified C_KIT-6_ samples are henceforth referred to as C_KIT-6_-*X*-*Y*-*Z*, where *X* denotes plasma time in min, *Y* plasma power in W, and *Z* pressure in the plasma chamber in mbar, giving the combinations presented in Table 1.

**Table 1:** Combinations of Plasma Treatment Parameters to Obtain Differently Oxidized C_KIT-6_

N^o^	time (–1)—6 s,(1)—15 min	power (–1)—40 W,(1)—100 W	pressure (–1)—0.2 mbar,(1)—0.8 mbar
1	–1	–1	1
2	–1	1	1
3	1	–1	1
4	1	1	1
5	–1	–1	–1
6	–1	1	–1
7	1	–1	–1
8	1	1	–1

#### Adsorption Tests of Co^2+^

To determine the sorption capacity of carbon materials toward Co^2+^, the adsorption of metal cations from the liquid phase was carried out. First, Co^2+^ solution (1500 mg L^–1^) was prepared via dissolving cobalt(II) perchlorate in the acetic acid buffer of pH 5. Then, 20 mg of each carbon sample was suspended in 40 mL of Co^2+^ solution and agitated (200 rpm) in the IKA KS 4000i control shaker for 24 h at room temperature. Afterward, materials were separated and collected for further analysis. To determine the exact concentration of Co^2+^ before adsorption (*C*_0_) and after adsorption (*C*_e_), the solutions were analyzed using flame atomic absorption spectrometry. In order to quantify the amount of adsorbed Co^2+^ on the carbon materials (*q*_e_), the following formula was applied.
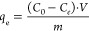
1where *C*_0_—initial Co^2+^ solution concentration (mg L^–1^), *C*_e_—the Co^2+^ solution concentration (mg L^–1^) remaining after the adsorption process, *V*—the volume of Co^2+^ solution used (L), and *m*—mass of the carbon adsorbent (g).

#### Deposition of Cobalt Phase

To obtain supported catalysts, deposition precipitation is often used. To deposit metal hydroxides, the pH of the solution is changed. The support present in the suspension acts as a stabilizer for the nuclei by reducing their surface free energy or for the precipitate by decreasing the energy barrier for nucleation. Thus, under some well-defined conditions, nucleation and deposition can occur only on the support, without precipitation in the bulk of the solution.^47^ In our case, a slow increase in pH would invariably change the surface properties of the carbon, and any correlation with the adsorptive properties tested in pH 5 buffer would be lost. Therefore, we decided to wash the carbons filtered after the adsorption test with 0.1 mol L^–1^ KOH in an attempt to precipitate the adsorbed Co^2+^ cations as Co(OH)_2_. After the deposition precipitation, the samples were dried at 60 °C for 12 h. C_KIT-6_-*X*-*Y*-*Z* samples with deposited cobalt oxide are henceforth referred to as C_KIT-6_-*X*-*Y*-*Z*-Co.

The elemental composition of the C_KIT-6_ samples with deposited cobalt oxide was examined using an X-ray fluorescence spectroscopy (XRF) with ARL Quant’X, Thermo Fisher spectrometer [4–50 kV, 1 kV step, Rh anode, 3.5 mm Si(Li) drifted crystal with a Peltier cooling (∼185 K) detector]. The quantitative analysis of cobalt was done with Uniquant software.

### 2.2 Electrochemistry

A Biologic BP-300 bipotentiostat connected to a Biologic RC-10K rotator was used to record all electrochemical measurements. The rotation rate was kept at 1600 rpm for all experiments. A glassy carbon electrode (GCE) was used as the rotating disk electrode (RDE) with a surface diameter of 3 mm. Before each experiment, the RDE was polished by using an aluminum oxide slurry (0.05 μm). We used a Hg/HgO electrode filled with NaOH 1.0 mol L^–1^ solution as the reference electrode and a platinum wire as an auxiliary electrode. All applied potentials were recalculated to the potentials of the reference hydrogen electrode (RHE) by using the measured pH according to eq 2.
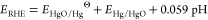
2where *E*_HgO/Hg_^Θ^ = 0.098 V versus normal hydrogen electrode, *T* = 25 °C.

All measurements were done in KOH 0.1 mol L^–1^ solution. The electrolyte solution was saturated with argon gas for 45 min before starting the measurements, and the electrochemical cell was flushed with argon also during the experiments.

To prepare ink for deposition onto the electrode, 2 mg of every catalyst was suspended in 0.375 mL of water, 0.125 mL of isopropanol, and 25 μL of Nafion 5% solution, and left in an ultrasound bath for 30 min. 6.6 μL of the prepared ink was pipetted dropwise on the GCE surface to obtain a 365 μg cm^–2^ catalyst loading. Every drop was dried at 200 rpm, and the last drop was left to dry under rotation for 45 min. The thin layer of catalyst film on GCE was stabilized electrochemically before the measurements started. To this end, we applied cyclic voltammetry (CV) scanning at a potential range of 0.2–0.9 V versus RHE with 10 cycles with a 100 mV s^–1^ scan rate, 10 cycles with a 20 mV s^–1^ scan rate, and 5 cycles with a 10 mV s^–1^ scan rate. Then, to determine double-layer capacitance, a series of CV scans were collected at a potential range of 1.1–1.2 V versus RHE, with the scan rates from 2 to 12 mV s^–1^. Chronoamperometric (CA) measurements were performed in the interval potential of 1.43–1.83 V versus RHE with 50 mV increment with the step for 15 min. The ohmic drop was corrected by using the averaging uncompensated resistance recorded before and after CA measurements, determined by impedance measurement at high frequencies (ZIR technique in EC-Lab software).

### 2.3 X-ray Photoelectron Spectroscopy

Powdered samples were used for XPS measurements. The XPS spectra were collected with a SESR4000 analyzer (Gammadata Scienta) in a vacuum chamber with a base pressure below 5·10^–9^ mbar. Monochromatized Al Kα source was used with 250 W at 1486.6 eV emission energy. The pass energy for selected narrow-range binding energy scans was 100 eV. CasaXPS Version 2.3.24PR1.0 was used to process the raw data.^48^ Binding energy scales were corrected for gold work function (WF) determined in the spectrometer, 4.65 eV.

### 2.4 Transmission Electron Microscopy

The catalysts were ground in an agate mortar into fine powders for the microscopic observations. The resulting powder of each sample was poured with 99.8% ethanol (POCH) to form a slurry which subsequently was inserted into an ultrasonic homogenizer for 20 s. Then, the catalyst-containing slurry was pipetted and supported on a 200-mesh copper grid covered with lacey formvar and stabilized with carbon (Ted Pella Company) and left on filter paper for ethanol evaporation. The samples deposited on the grid were inserted into a single-tilt holder and moved to the electron microscope.

The high-resolution electron microscope Titan G2 60–300 kV (FEI Company) was used to study the samples. It consisted of a field emission gun, a monochromator, a three condenser lenses system, an objective lens system, an image correction (C_s_-corrector), high-angle annular dark field detector, and an energy dispersive X-ray spectrometer and a Tecnai electron microscope. Microscopic studies of the catalysts were carried out at an accelerating voltage of the electron beam of 300 kV.

The size and the shape of particles in the catalysts were determined by using high-resolution transmission electron microscopy (HR-TEM) imaging with fast-Fourier-transform (FFT). Phase separation was performed with the FFT by using masking available in the Gatan Digital Micrograph software package. On the basis of the FFT generated from HRTEM images, individual phases with various crystallographic orientations were identified. The measurements of the crystallite size of the separated active phase allowed us to determine the distribution of the crystallite size. The particle size distribution was obtained by measuring the diameter of about 100 particles. The average size of particles was calculated from eq 3.
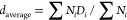
3where *N*_*i*_—the number of metal crystallites in a specific size range and *D*_*i*_—the average diameter in each diameter range.

## 3 Results and Discussion

### 3.1 Preliminary Characterization of Oxidized Carbon Supports (C_KIT-6_)

The as-synthesized ordered mesoporous carbon material (C_KIT-6_) was characterized by low-temperature nitrogen adsorption–desorption measurements, Raman spectroscopy, and thermogravimetric tests to evaluate the effect of plasma on the studied material. Neither short nor long plasma treatments do not modify the structure of the studied material. The initial material exhibited 827 m^2^ g^–1^ SSA, with a micropore surface of *S*_micro_ = 378 m^2^ g^–1^, the volume of the micropores *V*_micro_ = 0.36 cm^3^ g^–1^, and an average pore diameter of 5.8 nm. These values do not change after short (1 min) or long (10 min) oxygen plasma modification (100 W, 0.2 mbar O_2_). Oxidation with APS results in a decrease in SSA and associated porosity parameters (SSA = 656 m^2^ g^–1^, *S*_micro_ = 252 m^2^ g^–1^, *V*_micro_ = 0.25 cm^3^ g^–1^); however, the average pore diameter decreases only slightly to 5.5 nm (Table S1). Raman studies, which allow for a determination of the extent of graphitization changes of the material, confirmed the structural stability upon oxygen plasma treatment of C_KIT-6_. The ratio of the peaks assigned to disordered and graphitic fractions of carbon, *I*_D_/*I*_G_, does not change appreciably, even for 60 min of applied plasma (Figure S1A). However, despite the stability of the *I*_D_/*I*_G_ parameter, the C_KIT-6_ partially undergoes total oxidation in plasma and we observed a loss of the material. The thermal analysis further confirmed the stability of the plasma-modified carbon, and no changes in the thermal oxidation susceptibility after oxygen treatment (Figure S1B). To characterize surface electronic properties, we have determined the WF changes of the C_KIT-6_ after plasma oxidation and found that its value is not stable in time (Figure S1C,D). To stabilize the plasma-activated surface,^13^ we washed the carbon powder with deionized water and dried it at 60 °C in air. These results are in line with the oxidative plasma effect on the earlier studied graphene paper and graphite materials^12,13^ and indicate that plasma does not change the basic physical properties of the studied carbon material. Unless specifically stated otherwise, all further characterization results refer to the water-washed (stabilized) samples.

In contrast to physical properties, plasma oxidation always increases the concentration of surface oxygen groups, and the quantity depends primarily on the plasma treatment time. Interestingly, in contrast to the effect reported for graphene paper,^13^ washing with water decreases in the surface oxygen content of the plasma-oxidized carbon (Figure S2). Also, plasma oxidation always increases the electronic WF, as expected for the surface with electronegative oxygen species.^49^ The WF changes, oxygen atomic concentration determined from XPS analysis along with other properties of the reference, and plasma-modified C_KIT-6_ carbon are collected in Table 2. In general, the more surface oxygen, the higher the WF changes (Figure S3A), but some deviations from this trend can be noticed, as for the C_KIT-6_-15-100-0.2 sample.

**Table 2:** Basic Characterization Results of Reference and Plasma-Oxidized C_KIT-6_ Carbon

comments	sample name	WF changes/eV^a^	oxygen/at. %^b^	Co^2+^ sorption capacity/mg g^–1^	Co^2+^ sorption capacity/at nm^–2^	Co_3_O_4_/wt %^c^
no oxidation	C_KIT-6_-ref	0	3	17	0.21	0.38
plasma oxidation	C_KIT-6_-0.1-40-0.8	0.29	11	60	0.72	0.11
	C_KIT-6_-0.1-100-0.8	0.38	9	121	1.46	0.10
	C_KIT-6_-15-40-0.8	0.50	22	42	0.50	0.36
	C_KIT-6_-15-100-0.8	0.48	22	195	2.34	0.34
	C_KIT-6_-0.1-40-0.2	0.45	16	82	0.98	0.27
	C_KIT-6_-0.1-100-0.2	0.31	14	88	1.05	0.72
	C_KIT-6_-15-40-0.2	0.31	15	139	1.67	0.53
	C_KIT-6_-15-100-0.2	0.36	20	60	0.72	N/A
APS oxidation	C_KIT-6_-APS	N/A	13	163	1.96	0.43

a Versus C_KIT-6_-ref, water-washed, before adsorption.

b From XPS, before adsorption.

c From XRF, after equilibrium impregnation-precipitation.

Surface oxidation of carbon materials is well-known to increase their adsorptive properties toward transition metals, as described in the introduction section. Therefore, we performed adsorption tests with cobalt(II) perchlorate in pH 5 buffer to evaluate the adsorption capacity toward Co^2+^, *q*_Co_. The reference sample, wet-oxidized with APS exhibits an adsorption capacity similar to the reported nitric acid-oxidized C_KIT-6_ material.^18^ Plasma oxidation always increases *q*_Co_ as evidenced in Table 2. However, no direct correlation can be observed with the surface oxygen content derived for the XPS analysis (Figure S3B). To deposit the cobalt oxide phase on the oxidized mesoporous carbons, the precipitation with KOH after equilibrium adsorption was carried out on the filtered materials. After drying, elemental analysis with XRF was used to quantify the cobalt oxide content, and the results are also listed in Table 2. The cobalt phase was calculated as Co_3_O_4_ because that was the main phase evidenced by HR-TEM investigations, as shown in Section 3.4. This was somewhat surprising since we expected that the applied low-temperature drying would not transform the precipitated Co(OH)_2_ into Co_3_O_4_. Interestingly, the amount of deposited cobalt correlates neither with the surface oxygen content nor with the WF changes, as could be predicted for interactions with charged particles^50^ (Figure S3C,D). These results indicate that either the deposition procedure used does not properly account for specific surface interactions or the deposition process is more complicated than simple nucleation and growth of nanoparticles on surface adsorption sites.

### 3.2 Electrochemical Characterization

**Figure 1:**
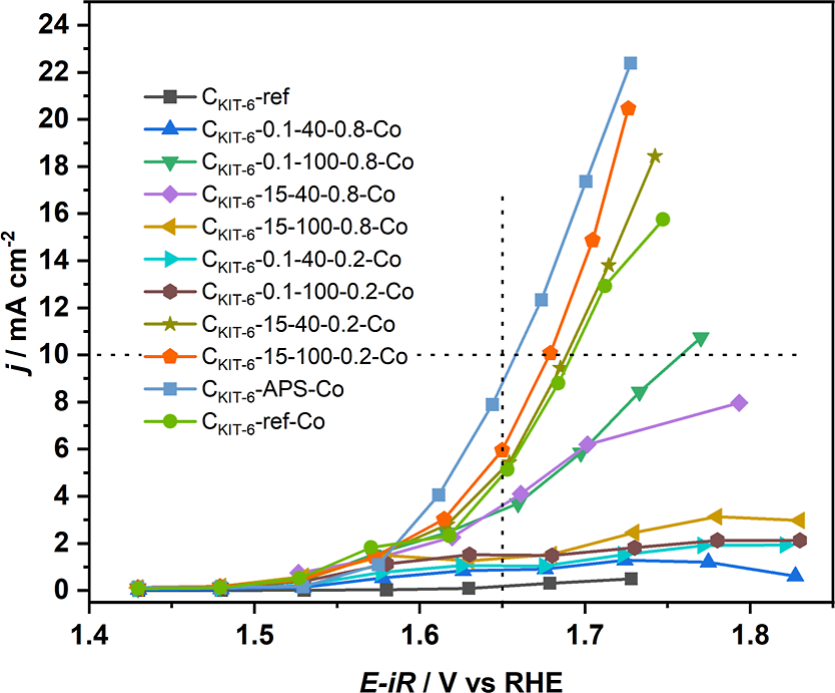
Electrocatalytic activity of cobalt oxide-doped oxidized C_KIT-6_ carbons toward the OER in a 0.1 M KOH solution.

To assess the activity in the OER of the studied plasma-oxidized ordered mesoporous carbon with deposited cobalt oxide active phase, CA tests were performed in 0.1 mol L^–1^ KOH using a rotating disc electrode. In addition to plasma-treated samples, the starting material (without cobalt oxide), C_KIT-6_ without oxidative pretreatment but with deposited cobalt oxide, and APS-oxidized sample with deposited cobalt oxide were also investigated (Figure S4). An interesting feature—oscillations of the current signal—for the C_KIT-6_-ref-Co sample can be observed in Figure S4. They are caused by the formation and removal of oxygen bubbles on the surface of the electrode. These oscillations are not present in the case of inactive (C_KIT-6_-ref) or oxidized samples. The decreasing activity and instability of the C_KIT-6_-APS-Co sample are also visible in Figure S4, where the disc current decreases over time at each potential step. The current values were determined by averaging the data from the last 10% of the step duration values, and the *iR* correction was applied based on the average resistance measured before and after the test. The resulting current versus potential graphs is presented in Figure 1.

The collected values of electrode current versus *iR*-corrected potential shown in Figure 1 reveal that in each case deposition of cobalt oxide causes an increase in activity compared to the C_KIT-6_-ref sample without cobalt oxide. However, some of the samples exhibit a negligible reactivity enhancement (e.g., C_KIT-6_-0.1-40-0.8-Co), and plasma pretreatment decreases the OER activity compared to the non-oxidized sample with deposited cobalt oxide (C_KIT-6_-ref-Co). The best samples achieve activity comparable to the pure cobalt oxide active phase (C_KIT-6_-15-100-0.2-Co, C_KIT-6_-APS-Co). The loading of Co_3_O_4_ is below 1 wt % for every modified C_KIT-6_ material (Table 2), which gives the total loading on the electrode of less than 3 μg. The observed best activity is similar compared to the 36 μg loading of Co(OH)_2_ and better than the 36 μg loading of Co_3_O_4_, as presented in Figure S5, where the activity of C_KIT-6_-APS-Co is plotted with Co(OH)_2_, Co_3_O_4_, and RuO_2_ reference materials at 36 and 365 μg loadings. We present the reactivity of the reference Co(OH)_2_ material because initially we did not expect the deposited cobalt phase to be Co_3_O_4_ spinel but rather Co(OH)_2_. Nonetheless, both types of materials exhibit comparable activity in OER.

Double-layer capacitance, *C*_DL_, or electrochemically active surface area, ECSA, is sometimes used to evaluate the reactivity of studied electrocatalysts. In our case, it is difficult to determine the specific capacitance, *C*_S_, of the electrode since two different components are present, carbon and Co_3_O_4_. Moreover, in the case of carbon-based composite materials, the carbon component usually controls the specific capacitance.^51^ However, since *C*_DL_ is directly proportional to ECSA (ECSA = *C*_DL_/*C*_S_), we calculated only the *C*_DL_ for the studied samples (data in Figure S6), and the results are presented in Table 3.

**Table 3:** Double-Layer Capacitance, *C*_DL_, and Anodic Current at 1.65 V vs RHE, *j*_1.65_, of Cobalt Oxide-doped C_KIT-6_ Carbons

comments	sample name	*C*DL/μF	*j*@1.65 V vs RHE (*j*_1.65_)/mA cm^–2^^a^
no oxidation	C_KIT-6_-ref	825	0.2
	C_KIT-6_-ref-Co	490	4.8
plasma oxidation	C_KIT-6_-0.1-40-0.8-Co	235	0.9
	C_KIT-6_-0.1-100-0.8-Co	820	3.4
	C_KIT-6_-15-40-0.8-Co	825	3.6
	C_KIT-6_-15-100-0.8-Co	885	1.4
	C_KIT-6_-0.1-40-0.2-Co	405	1.0
	C_KIT-6_-0.1-100-0.2-Co	730	1.5
	C_KIT-6_-15-40-0.2-Co	600	5.1
	C_KIT-6_-15-100-0.2-Co	955	5.9
APS oxidation	C_KIT-6_-APS-Co	1210	8.7

a Determined by a linear interpolation of data from Figure 1.

Plasma oxidation of mesoporous carbon emerged to be a method of choice to introduce surface oxygen groups while preserving the morphological features of the material. Although a prolonged plasma treatment may lead to the total oxidation of some carbon structures within the material, for the applied plasma parameters, both SSA and porosity are preserved. At the same time, the reactivity of the plasma-oxidized C_KIT-6_-15-100-0.2-Co sample is very close to the reactivity of the APS-oxidized C_KIT-6_-APS-Co sample and with better stability during chronopotentiometry tests (Figure S4). The decreasing reactivity of C_KIT-6_-APS-Co may be caused by changes in surface sulfates introduced during wet oxidation.^52^ For all oxidized samples, a smooth evolution of current in time can be observed, without sudden drops due to oxygen bubble formation. Therefore, the primary effect induced by the oxidation of the carbon support is a facilitation of gaseous oxygen release due to enhanced wettability of the surface.

A tentative analysis was performed to determine whether the observed electrocatalytic activity, expressed as a value of anodic current at 1.65 V versus RHE, correlates with the surface oxygen concentration, adsorption capacity, and deposited amount of Co_3_O_4_. The plots are presented in Figure S7; however, no correlation was observed. The presented results and preliminary analysis show that the activity in the OER of the studied samples is not a simple derivative of the support oxidation procedure or the amount of the deposited cobalt oxide active phase. Therefore, a deeper understanding of the surface features of the electrocatalysts is required to establish the relevant structure-reactivity correlations.

### 3.3 X-ray Photoelectron Spectroscopy

**Figure 2:**
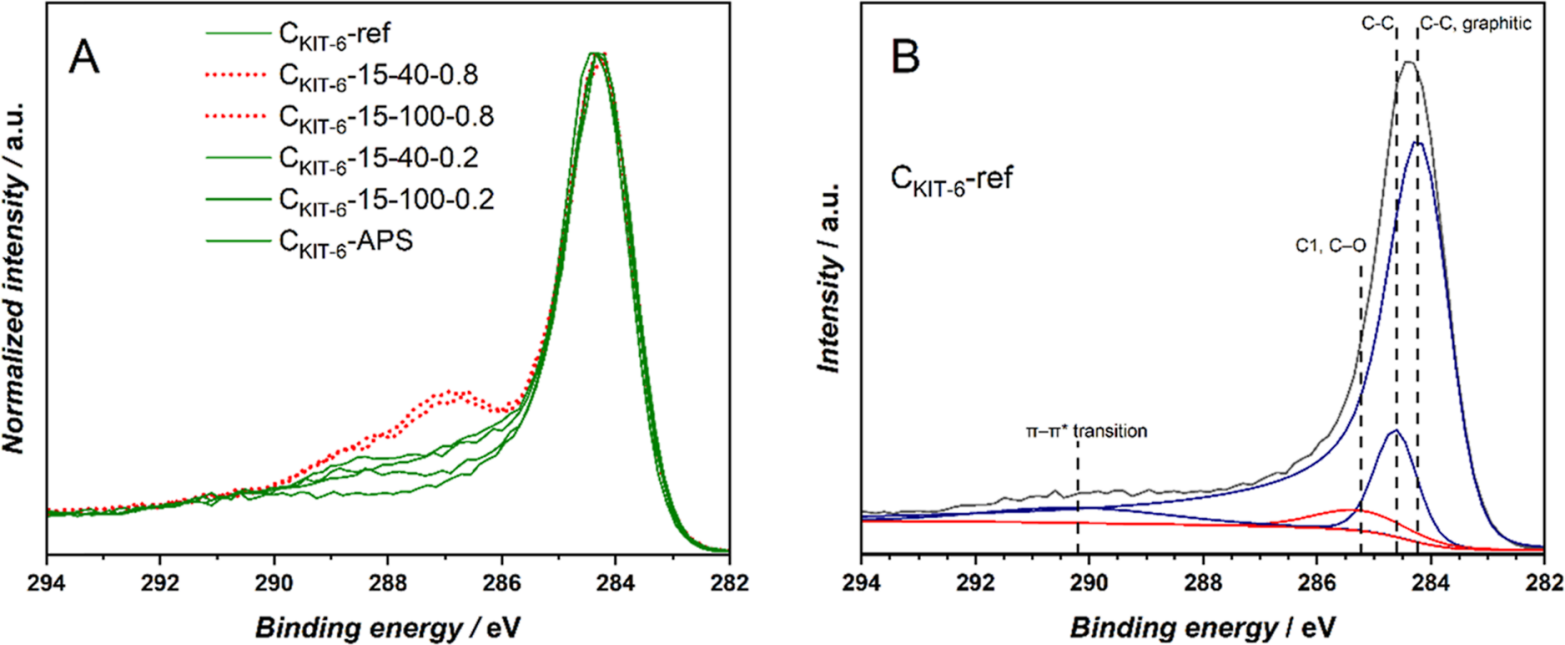
(A) Qualitative comparison of normalized XPS C 1s intensity of active (green full line) and inactive (red dotted line) cobalt oxide-doped C_KIT-6_ materials. (B) Curve-fitting of XPS C 1s spectrum of C_KIT-6_-ref used as a model for oxidized samples.

To further investigate the surface properties of the C_KIT-6_ samples oxidized with plasma (before cobalt oxide deposition), we focused on the XPS analysis of the C 1s binding energy range. In qualitative terms, the electrocatalytically inactive samples exhibit a relatively more pronounced peak at 287.1 eV, which is usually assigned to C=O in carbonyl groups and carbons attached to two ether/hydroxyl groups,^53^ shown in Figure 2A with red, dotted line. At the same time, for the samples which exhibit pronounced reactivity this peak is not so intense, and these samples usually have an increased intensity at 288.4 eV assigned to COO in carboxyl, lactone, and ester groups.^53^ This group is presented in Figure 2A with a green line. To quantify the effect of speciation of oxygen functional groups, we performed curve-fitting for the C 1s spectral range for all investigated mesoporous carbon supports.

Since curve-fitting of any photoelectron peak is a challenging process, especially so for carbon 1s,^54^ the reference C_KIT-6_ material with a minimal number of components was fitted first. This approach accurately reflected the low oxygen concentration (Table 2), as shown in Figure 2B, but any information on the minor C=O and COO components was lost. The main feature was fitted with an asymmetric component of graphitic conductive carbon at 284.1 eV and another C–C band at 284.6 eV. These two peaks are complemented by a broad band assigned to an aromatic shake-up peak due to π–π* transition centered above 290 eV. The remaining band (marked in red in Figure 2B) at 285.3 eV has to be then assigned to C–O type surface oxygen groups, despite relatively low peak center energy.^53,54^ This set of curves served as a model for fitting plasma and APS-oxidized samples.

**Figure 3:**
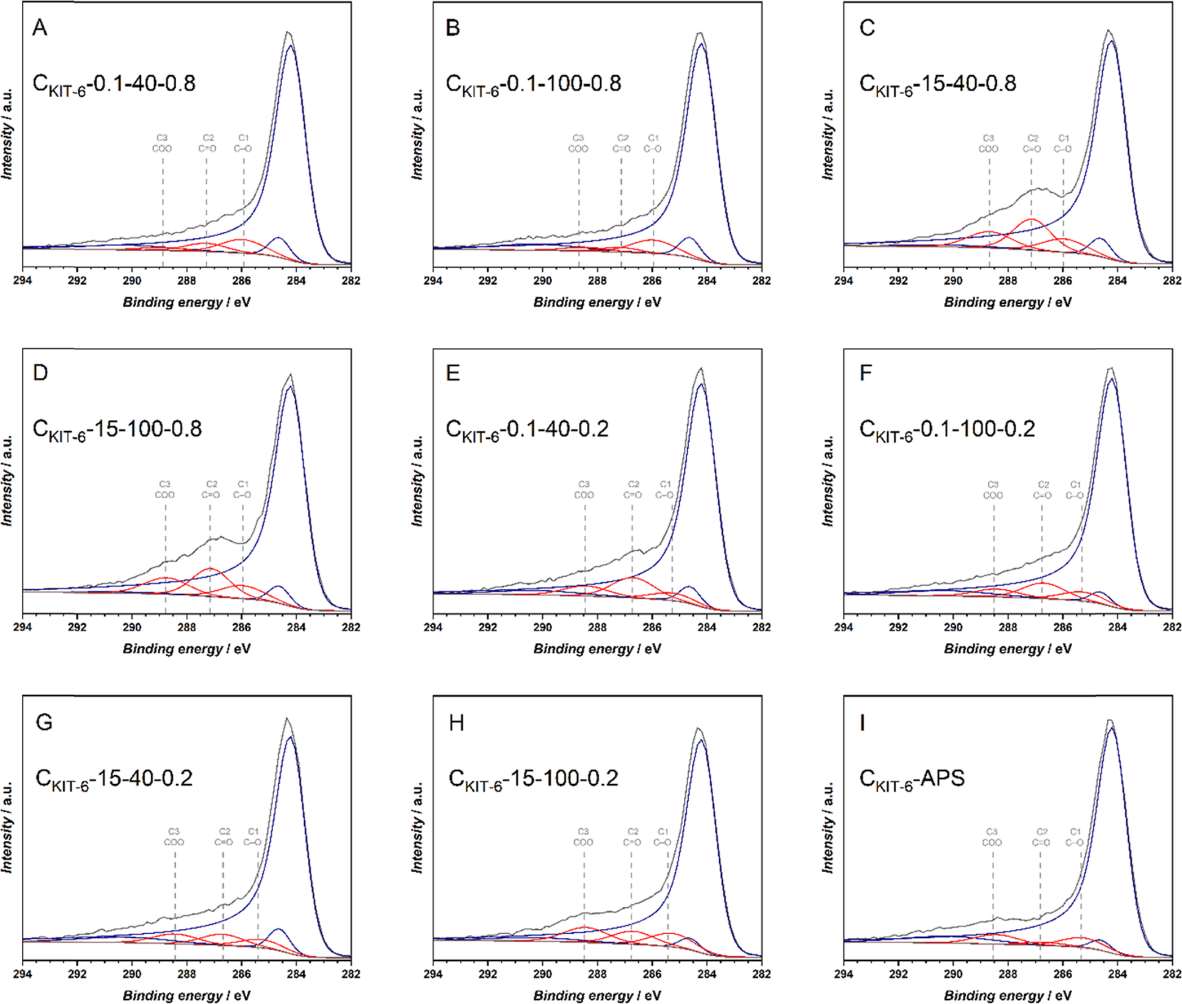
Curve-fitting of C 1s XPS of APS- and plasma-oxidized C_KIT-6_ samples (A) C_KIT-6_-0.1-40-0.8, (B) C_KIT-6_-0.1-100-0.8, (C) C_KIT-6_-15-40-0.8, (D) C_KIT-6_-15-100-0.8, (E) C_KIT-6_-0.1-40-0.2, (F) C_KIT-6_-0.1-100-0.2, (G) C_KIT-6_-15-40-0.2, (H) C_KIT-6_-15-100-0.2, and (I) C_KIT-6_-APS.

The peak positions and full widths at half maximum from the model in Figure 2B were applied as a constant feature of all subsequent spectra and added peaks due to the formation of oxygen groups of the C=O and COO types, as shown in Figure 3C,H, respectively. Such a curve-fitting procedure yielded consistent positions and widths of the components and allowed quantifying the contributions of the different families of oxygen functional groups to the total oxygen content. To verify the validity of this approach, the surface concentration of oxygen based on the C 1s components was calculated, assuming that there is one oxygen atom per C–O type group, one oxygen atom per C=O type group, and two oxygen atoms per COO type groups. This is a simplified approach but enabled validation of whether the curve fitting yielded reasonable values. The C 1s component peak areas and the verification with the described model are collected in Table S2. The O 1s spectral range for modified C_KIT-6_ carbons varies in intensity due to different surface oxygen concentrations (Figure S8A). Normalization of the O 1s bands reveals no substantial shifts in the relative intensities of the different surface oxygen species (Figure S8B), which makes this spectral region not suitable for quantification of their contributions in different samples.

**Figure 4:**
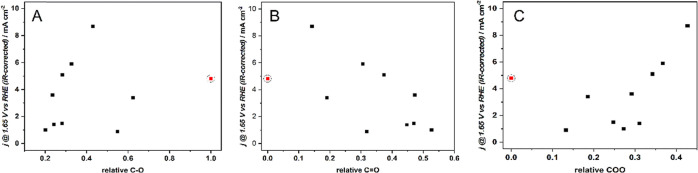
Correlation between anodic current at 1.65 V vs RHE (*iR*-corrected) of OER, *j*_1.65_, and the relative content of (A) C1, C–O-type groups, (B) C2, C=O-type groups, and (C) C3, COO-type groups. The encircled red point designates the C_KIT-6_-ref sample, where the C=O- and COO-type groups were not determined.

Speciation analysis based on curve-fitting of C 1s spectra presented in Figure 3 allowed for the verification of the observation from Figure 2A about the negative impact of C=O type groups on the OER reactivity of C_KIT-6_ materials with the deposited cobalt oxide phase. The interpolated electrode current from Table 3, *j*_1.65_, is plotted against the surface concentration of oxygen groups determined from the C 1s spectral range from Table 4 and shows no correlation for any of the oxygen groups, as presented in Figure S9. Further analysis of the relative contribution of each component (Table S2) suggested no correlation with C–O type groups (C1), a negative correlation between the C=O type groups (C2) and the OER activity, and a positive correlation between COO type groups (C3) and the OER activity; see Figure 4. A similar analysis of plotting other C_KIT-6_ properties from Table 2 against individual contributions to the C 1s band from surface oxygen group types does not yield any satisfactory correlations.

**Table 4:** Oxygen Speciation on Oxidized C_KIT-6_ Carbons Based on Curve-Fitting of XPS C 1s Spectra

sample name	C–OC1/at. %	C=OC2/at. %	COO C3/at. %	C1 + C2 + 2·C3^a^	experiment model^b^	C3/(C2 + C3), *x*_COO_
C_KIT-6_-ref	4.1			4	0.9	
C_KIT-6_-0.1-40-0.8	5.8	3.4	1.4	12	0.5	0.294
C_KIT-6_-0.1-100-0.8	5.7	1.7	1.7	11	0.7	0.494
C_KIT-6_-15-40-0.8	4.8	9.7	6.0	26	3.2	0.381
C_KIT-6_-15-100-0.8	4.8	8.8	6.1	26	2.9	0.410
C_KIT-6_-0.1-40-0.2	2.9	7.7	4.0	19	1.0	0.341
C_KIT-6_-0.1-100-0.2	3.6	5.9	3.1	16	1.0	0.345
C_KIT-6_-15-40-0.2	3.2	4.3	3.9	15	0.5	0.478
C_KIT-6_-15-100-0.2	5.5	5.1	6.2	23	0.3	0.546
C_KIT-6_-APS	4.0	1.3	4.0	13	–0.4	0.750

a The simplest model accounting for the number of oxygen atoms bonded to carbon atoms resulting in component to C 1s band.

b Experimental values from area_O1s_(area_O1s_ + area_C1s_) presented in Table 2.

The validity of the adopted approach can be rationalized by an analysis of the contribution of oxygen groups on the WF changes. Taking the WF changes from Table 2 as a linear combination of the three main types of oxygen groups with their surface atomic concentration as a weight (C1, C2, and C3, Table 4), one can determine the values proportional to effective surface dipole moment (*a*, *b*, *c*), ΔWF = *a*·C1 + *b*·C2 + *c*·C3. The results are presented in Figure S10. Linear regression yields values *a* = 4.1, *b* = 3.3, and *c* = 0.0 for C1, C2, and C3 type groups, respectively. They correlate directly with values calculated for such oxygen groups on graphenic sheets, where the dipole moment projection in the normal direction was −0.58 D for −OH, −0.33 and −0.93 D for −CHO, and C=O and 0.13 D for −COOH type groups.^55^

**Figure 5:**
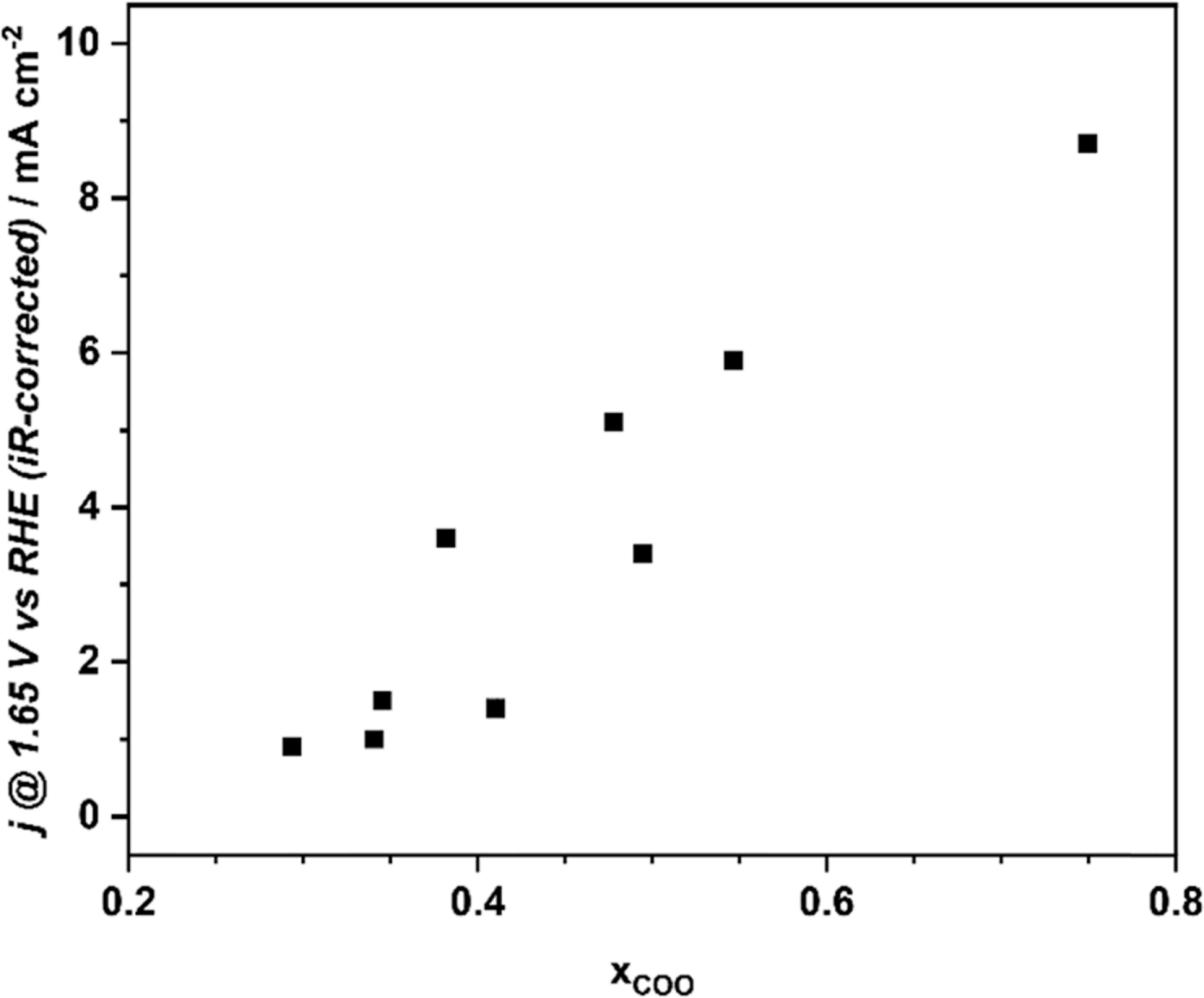
Correlation between anodic current at 1.65 V vs RHE (*iR*-corrected) of OER, *j*_1.65_, and the content of COO-type groups relative to C=O-type groups, x_COO_.

**Figure 6:**
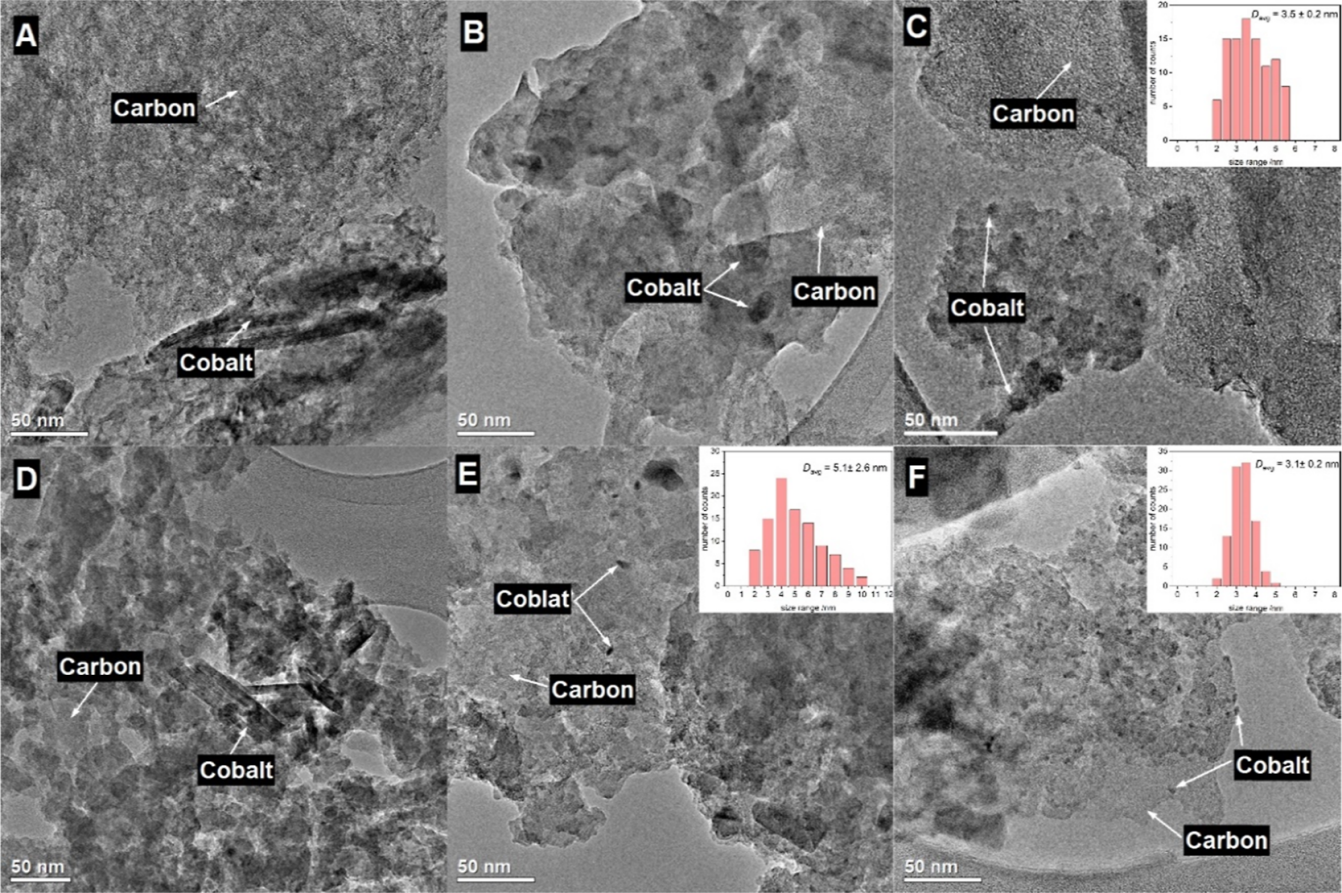
TEM pictures of (A) C_KIT-6_-ref-Co, (B) C_KIT-6_-0.1-40-0.8-Co, (C) C_KIT-6_-0.1-100-0.8-Co, (D) C_KIT-6_-0.1-100-0.2-Co, (E) C_KIT-6_-15-40-0.2-Co, and (F) C_KIT-6_-APS-Co with the cobalt oxide size distribution for selected samples (insets in pictures C,E,F).

The correlation between the electrocatalytic activity and speciation of oxygen groups shown in Figure 4C is much more obvious when the electrode current *j*_1.65_ is plotted against the relative distribution of only COO and C=O components, x_COO_ = C3/(C2 + C3), as presented in Figure 5. It shows that the reactivity of samples containing cobalt oxide active phase deposited onto the C_KIT-6_ increases with the relative number of COO-type surface groups versus C=O-type groups. As evidenced by the reactivity tests and microscopic characterization, it is the quality not the quantity of the oxygen functional groups which controls the adsorption of cobalt ions (Table 2), dispersion of the formed cobalt oxide phase (Figure 6), and electrocatalytic activity of the cobalt oxide/C_KIT-6_ composite materials (Figure 1). Two main effects may contribute to the observed relationship *j*_1.65_ versus x_COO_, enhanced dispersion of Co_3_O_4_, and participation of the surface oxygen groups in the reaction. First, the improved reactivity may be caused by a better dispersion of Co_3_O_4_ on the support as shown in the TEM images for the selected samples. Then, the resultant reactivity would be a combination of the Co_3_O_4_ quantity and its dispersion. For the sample pairs selected for TEM observations, the more active C_KIT-6_-0.1-100-0.8-Co and C_KIT-6_-15-40-0.2-Co also exhibit better dispersion than their less active counterparts C_KIT-6_-0.1-40-0.8-Co and C_KIT-6_-0.1-100-0.2-Co. Similarly, C_KIT-6_-0.1-100-0.8 and C_KIT-6_-15-40-0.2 exhibit higher adsorption capacities than C_KIT-6_-0.1-40-0.8 and C_KIT-6_-0.1-100-0.2. In the same line of thought, the C_KIT-6_-APS sample exhibits one of the highest adsorption capacities and the highest x_COO_ value. After the deposition of Co^2+^ ions on this sample, the cobalt oxide is very well-dispersed and exhibits the highest electrocatalytic activity.

The second effect contributing to the observed linear *j*_1.65_ versus x_COO_ relationship may be the participation of carbon with oxygen functional groups in the catalytic OER. It was suggested experimentally^56^ and supported by quantum chemical calculations^57^ that the C=O-type oxygen functional groups contribute the most to OER over metal-free oxidized carbons. However, a recent study argues that the presence of carboxylic groups is most beneficial for enhanced OER activity of double-walled carbon nanotubes.^58^ Furthermore, when a metal oxide phase is introduced, interactions of the material components will occur at their interface and may contribute to the formation of new active centers. It was recently shown that over CoO_*x*_ catalyst, the third OER step is probably the rate-determining step, M-OOH + OH^–^ → M-OO + H_2_O + e^–^.^59^ However, studies of cobalt oxyhydroxide (CoOOH) as the active phase led to the identification of the release of dioxygen from the superoxide intermediate (Co–O–O^•^–Co) as the rate-determining step of the OER (fourth step).^60^ In such a case, the carbon support may enhance the reaction by means of the oxygen spillover mechanism, as previously reported for carbon-perovskite oxygen evolution catalysts.^41^

As evidenced by the XPS results, plasma oxidation may introduce different types of oxygen functionalities. The two most active plasma-modified C_KIT-6_ samples, C_KIT-6_-15-40-0.2-Co and C_KIT-6_-15-100-0.2-Co, share common characteristics in terms of plasma treatment parameters: long plasma time and low oxygen pressure in the chamber. Furthermore, the most active C_KIT-6_-15-100-0.2-Co sample was prepared by pretreatment with the highest plasma generator power. A natural conclusion is that to effectively oxidize the carbon surface for the subsequent deposition of the active phase, rather harsh plasma conditions should be used. However, the applied 15 min of plasma treatment resulted in substantial total oxidation of the carbon material (more than 50%). In addition, different plasma systems are available, varying in such fundamental aspects as plasma generation type (capacitive and inductive radio-frequency discharges) and resulting critical operational parameters. Therefore, careful optimization of not only the plasma treatment time but also other parameters (e.g., generator power, gas pressure) should be performed because these parameters are specific to the particular plasma system. Regardless of the plasma system used, maximization of the carboxylic-type oxygen groups should be a priority for effective pretreatment of the carbon support toward oxide phase deposition.

### 3.4 Transmission Electron Microscopy

**Figure 7:**
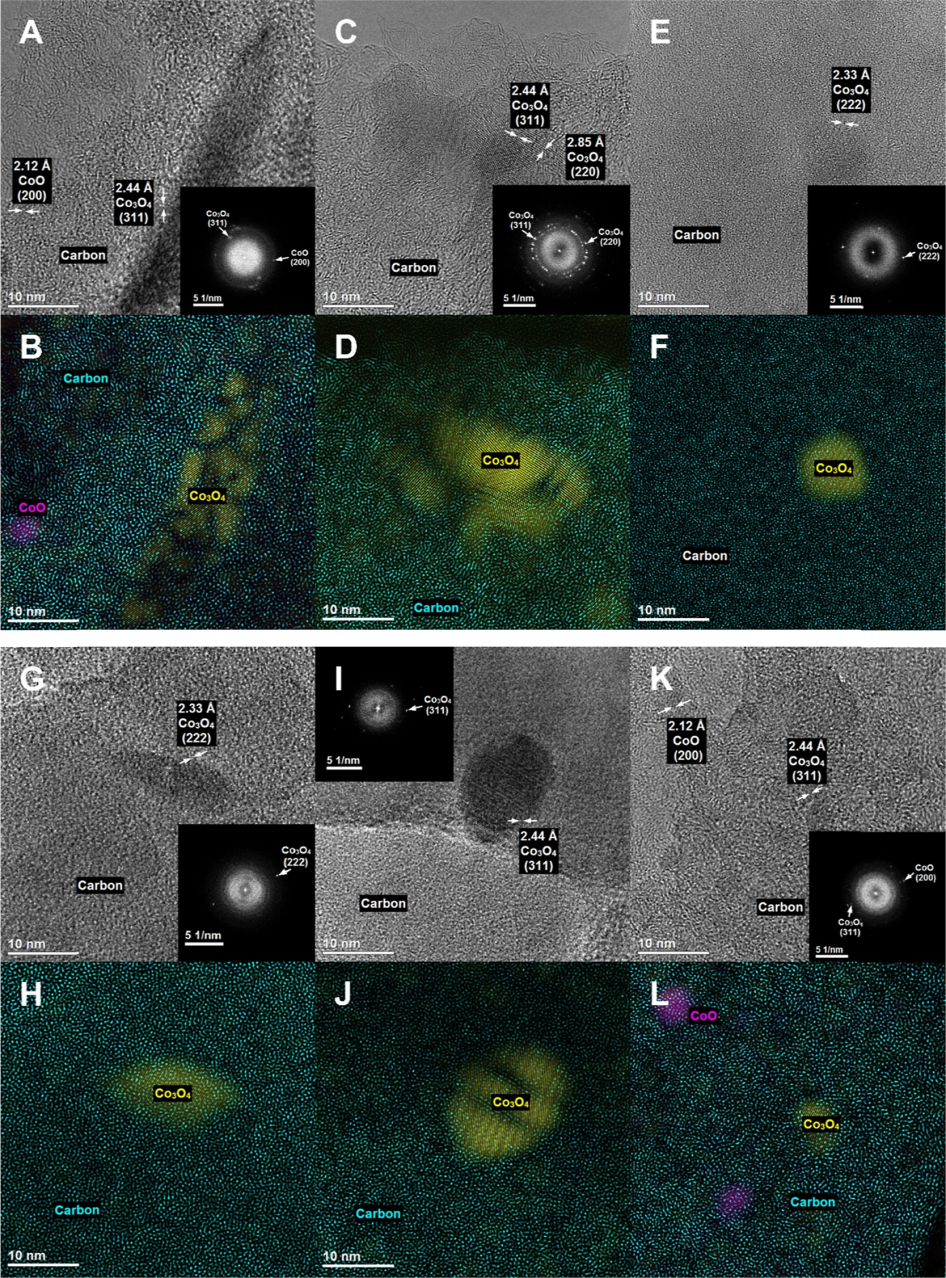
HR-TEM pictures of (A,B) C_KIT-6_-ref-Co, (C,D) C_KIT-6_-0.1-40-0.8-Co, (E,F) C_KIT-6_-0.1-100-0.8-Co, (G,H) C_KIT-6_-0.1-100-0.2-Co, (I,J) C_KIT-6_-15-40-0.2-Co, and (K,L) C_KIT-6_-APS-Co.

The microscopic characterization studies aimed to evaluate the dispersion (Figure 6) and composition (Figure 7) of the cobalt oxide phase on the selected plasma-oxidized mesoporous carbon supports. We used TEM analysis at different magnifications to obtain information about the population of the nanoparticles and individual nanocrystals. All samples chosen for this study contained the deposited cobalt oxide active phase and included the reference C_KIT-6_-ref, material oxidized with APS, as well as two pairs of plasma-oxidized carbons. Each pair of the plasma-oxidized carbons contained a similar amount of cobalt oxide, as determined by XRF (Table 2) in the range of 0.1 wt % (C_KIT-6_-0.1-40-0.8-Co and C_KIT-6_-0.1-100-0.8-Co) and 0.6 wt % (C_KIT-6_-0.1-100-0.2-Co and C_KIT-6_-15-40-0.2-Co). In each of the pairs of plasma-oxidized carbons, the two samples exhibited very distinct electrocatalytic activity (Table 3), despite the similar cobalt oxide content.

From the obtained TEM images (Figure 6) it can be deduced that for the samples C_KIT-6_-ref-Co, C_KIT-6_-0.1-100-0.8-Co, C_KIT-6_-15-40-0.2-Co, and C_KIT-6_-APS-Co the cobalt p oxide phase is present in a rather well-dispersed state, whereas for C_KIT-6_-0.1-40-0.8-Co and C_KIT-6_-0.1-100-0.2-Co, agglomerates are predominant. In the samples with well-dispersed nanocrystals, the mean crystallite size is in the range of 3–5 nm (inserts in Figure 6C,E,F). The C_KIT-6_-ref-Co sample contains additional, larger elongated crystals. Additional TEM images of the selected materials are presented in Figure S11. The cobalt oxide is present mainly in the form of spinel Co_3_O_4_, but some CoO crystals are also evidenced (Figure 7). These results reveal that the dispersion of the cobalt oxide active phase is correlated with the activity in electrochemical OER over the studied materials, where the materials with the active phase in a dispersed state are much more active than those containing agglomerates. It is not surprising that the effect of more effective usage of the well-dispersed active phase is well-known in the studies of heterogeneous catalysts.

The cobalt oxide active phase deposited onto the non-modified C_KIT-6_-ref appears to be not very well-dispersed, with the presence of elongated crystals (Figure 6A). At the same time, the oxygen content as seen from the XPS is very low, only 3 at. %, which suggests that, to some extent, the wettability or the surface concentration of oxygen groups (not relative speciation) may be equally important for the Co_3_O_4_ dispersion. However, the catalytic activity of this material is surprisingly high. The curve-fitting analysis of oxygen groups in the C 1s spectral range of C_KIT-6_-ref is impossible due to the very low intensity of the components other than C–O-type (Figure 2B). One explanation for the high activity of the C_KIT-6_-ref-Co sample is that it contains a substantial number of acidic surface groups (2.5 mmol g^–1^), much more than basic groups (0.3 mmol g^–1^), as determined by Boehm titration (details in Supporting Information, Appendix A). These groups likely exhibit similar characteristics to the COO-type groups introduced by plasma and APS oxidation since the APS oxidation increases acidic groups on C_KIT-6_ and removes the basic groups. Plasma oxidation, on the other hand, depending on the parameters, may decrease the concentration of acidic groups and increase the concentration of basic ones (Table S3).

Cooperative action of the carbon support and the cobalt oxide phase can be also inferred from the evaluation of the activity of oxidized carbons without deposited Co_3_O_4_, C_KIT-6_-15-100-0.2, and C_KIT-6_-APS. Both materials exhibit increased OER activity compared to C_KIT-6_-ref material (Figure S12A); however, the stability under the reaction conditions is low (Figure S12B). Interestingly, the observed OER activity is in several cases higher than that for other C_KIT-6_ samples with deposited cobalt oxide phase, which may indicate the important role of the support–active phase interaction.

The presented results point to the tailored oxidation of carbon supports to enhance active phase dispersion and increase the overall OER activity as a viable strategy for catalyst development. Moreover, the presented findings also point to new research directions, such as studies on the possible synergistic effect of carbon support and metal oxide active phase. A question related to this effect is whether the diffusion of reaction intermediates can occur to enable synergy. Is the interphase diffusion of reaction intermediates possible? If there is synergy, what steps of the OER catalytic cycle on either component may be influenced? It becomes apparent that there is a need for complementary characterization of composite electrocatalysts under reaction conditions, that is, pH 13. Furthermore, a detailed description of the nucleation and deposition precipitation mechanism on carbon supports with different oxidation degrees and speciation of surface groups is necessary to optimize the loading of the active phase.

## 4 Conclusions

In this study, we aimed at evaluating the role of different surface oxygen groups on ordered mesoporous carbon on the amount and dispersion of the cobalt oxide active phase, which is a very popular material in many catalytic reactions, especially in electrocatalytic OER. We found that on the oxidized carbons, independently of the total surface oxygen content, it is the relative abundance of carboxylic-type oxygen groups that controls the dispersion and the activity of the cobalt oxide-carbon composite catalyst. The oxidative pretreatment does not directly influence the amount of the deposited active phase when using equilibrium adsorption-precipitation deposition of cobalt oxide. It was evidenced that the OER reactivity is controlled by the combined quantity and dispersion of the active phase, where the samples with the most dispersed cobalt oxide exhibit the highest activity. Moreover, we established a correlation between the relative abundance of COO-type surface groups and OER activity, which suggests a participation of the carbon support in the reaction, possibly through the spillover mechanism. The presented results indicate that to control the speciation of the deposited metal oxide phase on the carbon support particular attention must be paid to obtaining a surface with a high fraction of carboxylic type groups. The questions that need to be answered in the future are how does the oxygen speciation influence deposition of higher quantities of the active phase and what is the mechanism that couples the active phase with oxidized carbon in the OER?

## Supporting Information Available

Experimental details and results of thermogravimetric studies, Raman spectroscopy, measurements of the WF changes, Boehm titration, and additional spectroscopic (Raman, XPS), microscopic (TEM), and electrochemical characterization results (PDF)

## Notes

Deposited XPS data: 10.17632/49c4nhw7ch.1.
