# Supplementary material for: Speciation of Oxygen Functional Groups on the Carbon Support Controls the Electrocatalytic Activity of Cobalt Oxide Nanoparticles in the Oxygen Evolution Reaction
Source: ACS Appl Mater Interfaces. 2023 Jan 19;15(4):5148–60. doi: 10.1021/acsami.2c18403 (PMC9906611; doi:10.1021/acsami.2c18403)
Supplement: Supplementary file 1 — am2c18403_si_001.pdf [file am2c18403_si_001.pdf]

Supporting Information

Appendix A

Speciation of oxygen functional groups on the carbon support controls the electrocatalytic activity of cobalt oxide nanoparticles in the oxygen evolution reaction

Aleksander Ejsmont[a], Karolina Kadela[b], Gabriela Grzybek[b], Termeh Darvishzad[b], Grzegorz Słowik[c], Magdalena Lofek[b], Joanna Goscianska[a], Andrzej Kotarba[b], Paweł Stelmachowski[b]*

[a]Department of Chemical Technology, Faculty of Chemistry, Adam Mickiewicz University in Poznań, Uniwersytetu Poznańskiego 8, 61-614 Poznań, Poland

[b]Faculty of Chemistry, Jagiellonian University, Gronostajowa 2, 30-387 Krakow, Poland

[c]Department of Chemical Technology, Faculty of Chemistry, Maria Curie-Sklodowska University in Lublin, Maria Curie-Sklodowska Sq. 3, Lublin, 20-031, Poland

** pawel.stelmachowski@uj.edu.pl*

Experimental details

Thermogravimetric analysis of reference and functionalized samples was performed using TGA/DSC 1 equipment (Mettler Toledo). Approximately 10 mg of the sample were heated in the flow of synthetic air 40 ml·min^{-1} (mixed with Ar 20 ml·min^{-1}) in the temperature range of 25–1100 °C with a heating rate of 20 degrees per minute.

Raman spectroscopy was used to study local structural changes of oxidized carbons with a Renishaw InVia spectrometer equipped with a 514 nm laser. µRaman spectra were collected in the range of 1000–3000 cm^{-1} with a resolution of 1 cm^{-1}, by accumulating ten scans for each spectrum.

Changes in the work function of graphite samples were investigated by measuring contact potential difference (ΔCPD) measurements. The experiments were carried out using the Kelvin probe method with a KP6500 device (McAllister Technical Services). A stainless-steel plate (3 mm diameter) was used as an electrode (WF_{ref} = 4.3 eV). The measurements were carried out under ambient conditions (room temperature, atmospheric pressure), with vibration frequency at 114 Hz and amplitude at 40 a.u.

To establish the acid/base properties of the pristine and oxidized carbon materials **the Boehm titration method** was applied. The number of acidic oxygen functional groups was determined as follows. The 0.1 g of carbon material was suspended in 10 ml of 0.1 mol l^{-1} sodium hydroxide solution (POCH). Whereas, for the basic functional groups' analysis the carbon powders were flooded with a hydrochloric acid solution (10 ml, 0.1 mol l^{-1}, Chempur). Suspensions were then agitated for 24 h at RT, followed by materials separation. The residual solutions were lastly titrated in the presence of methyl orange as an indicator. 10 ml of each solution was taken, then titrated accordingly with a hydrochloric acid solution (0.1 mol l^{-1}) to establish acidic groups amount, and sodium hydroxide solution (0.1 mol l^{-1}) for basic groups.

Figures S1 – S12, Tables S1- S3

Table S 1 Porosity of reference and plasma modified ordered mesoporous carbon C_{KIT-6}.

Sample	SSA (m^2 g^{-1})	S_{micro} (m^2 g^{-1})	V_{micro} (cm^3 g^{-1})	D (nm)
C_{KIT-6}	827	378	0.36	5.8
C_{KIT-6} 1 min O_2 plasma	876	403	0.37	5.8
C_{KIT-6} 20 min O_2 plasma	817	418	0.37	5.8
C_{KIT-6}-APS	656	252	0.25	5.5

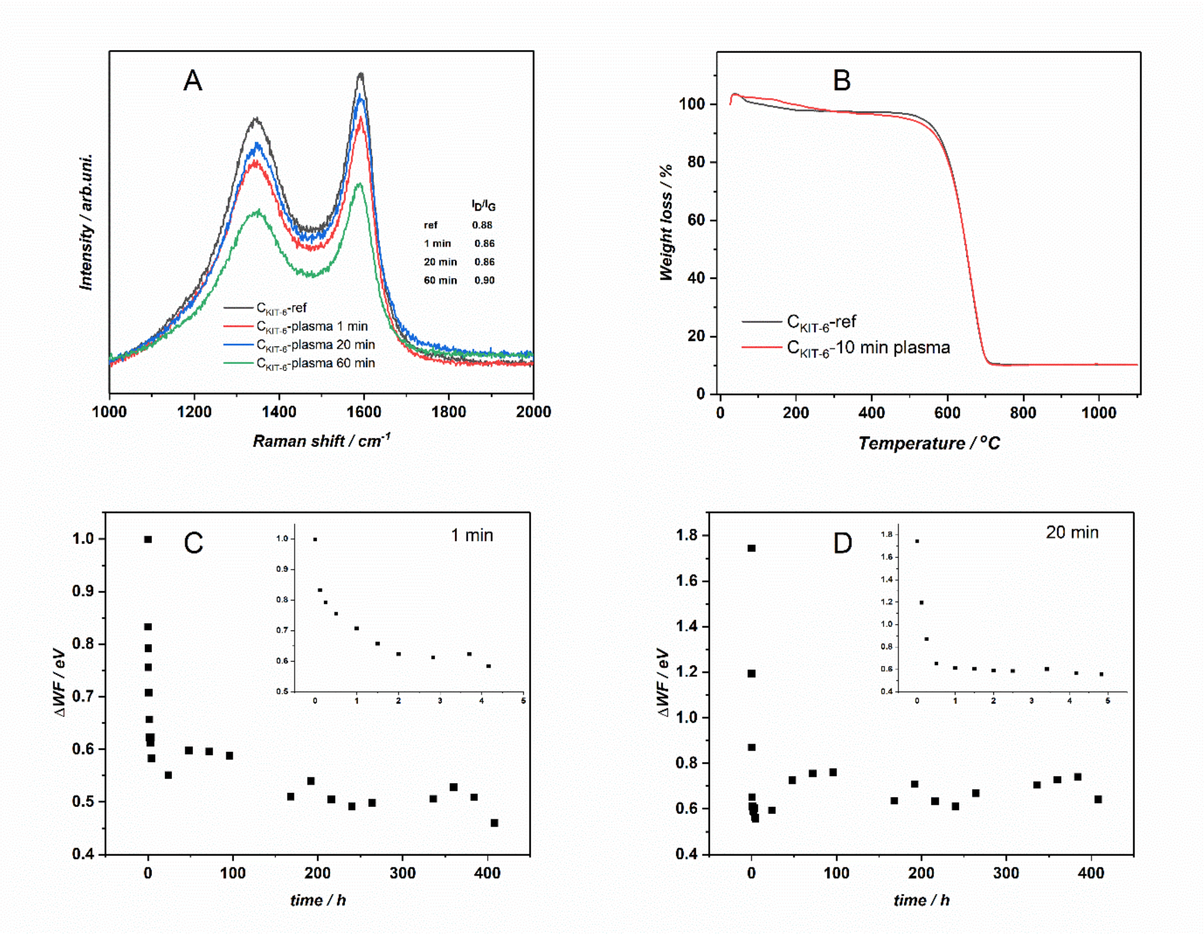

Figure S 1 Effect of plasma oxidation on the C_{KIT-6} mesoporous carbon. A) Raman spectra, B) thermogravimetric changes of oxidation in air, and wortk function changes after C) 1 min of plasma and D) 20 min of plasma.

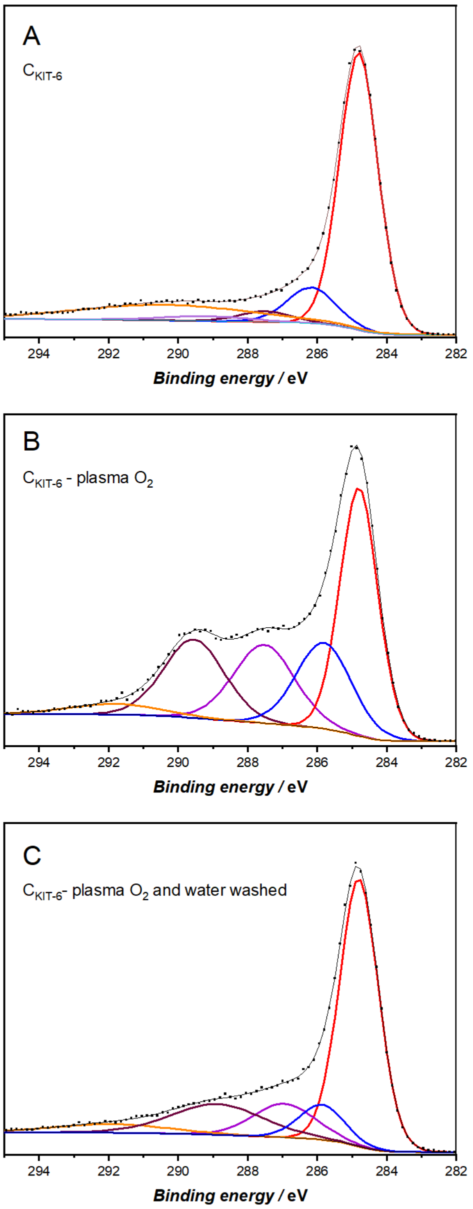

Figure S 2 XPS analysis of A) a reference (2 at.% O) B) fresh after plasma (30 at.% O), and C) water-washed C_{KIT-6} (15 at.% O). Oxygen plasma: 10 min, 100 W, 0.2 mbar.

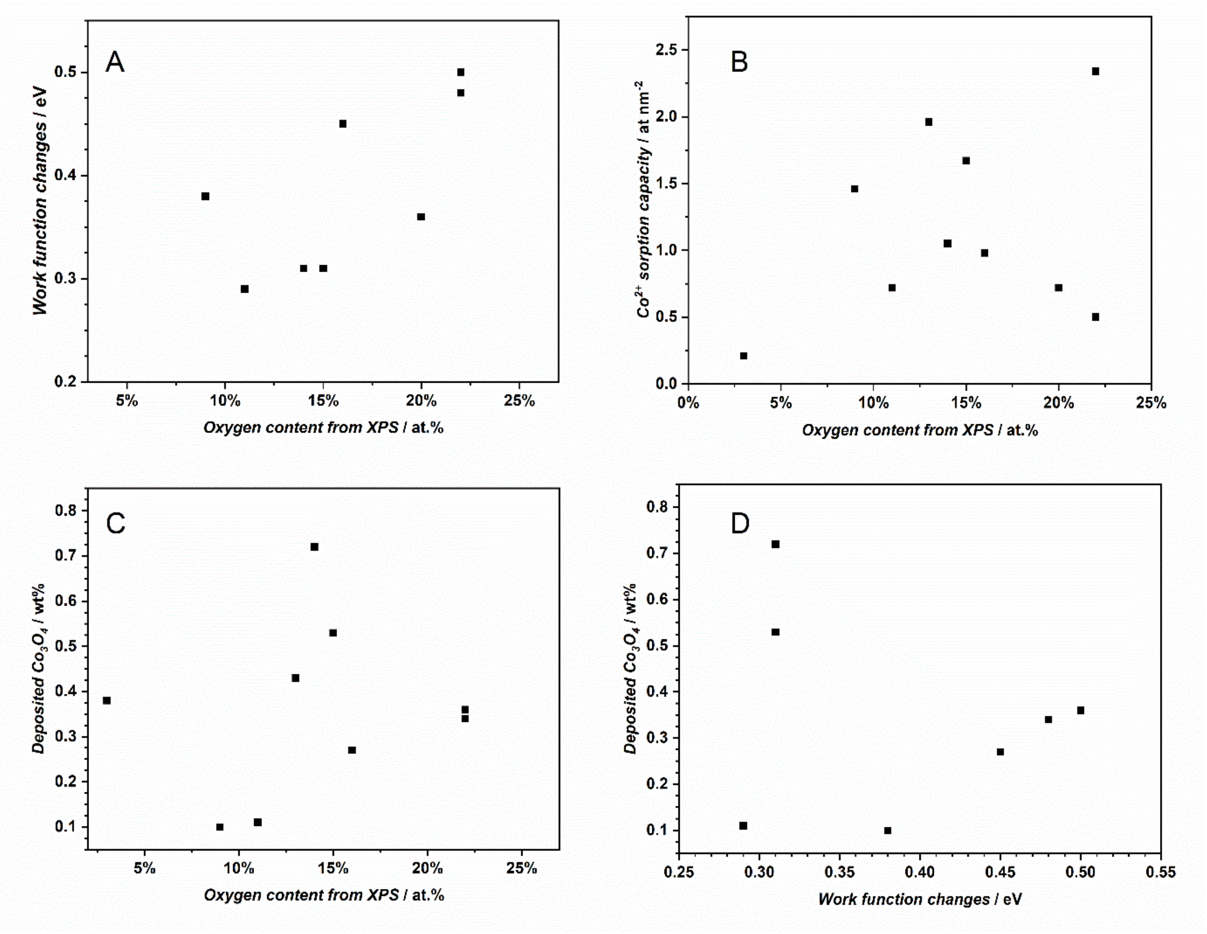

Figure S 3 Analysis of possible correlations between surface properties of modified C_{KIT-6} based on Table 2.

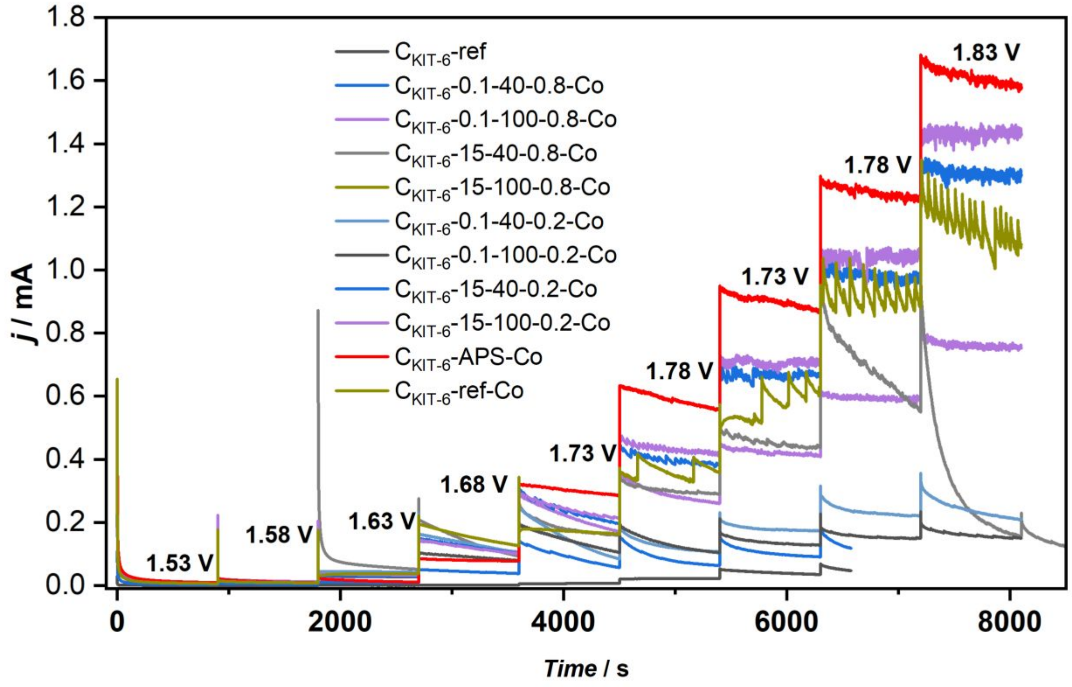

Figure S 4 Chronoamperometric results of oxygen evolution reaction studies on cobalt-doped C_{KIT-6} materials.

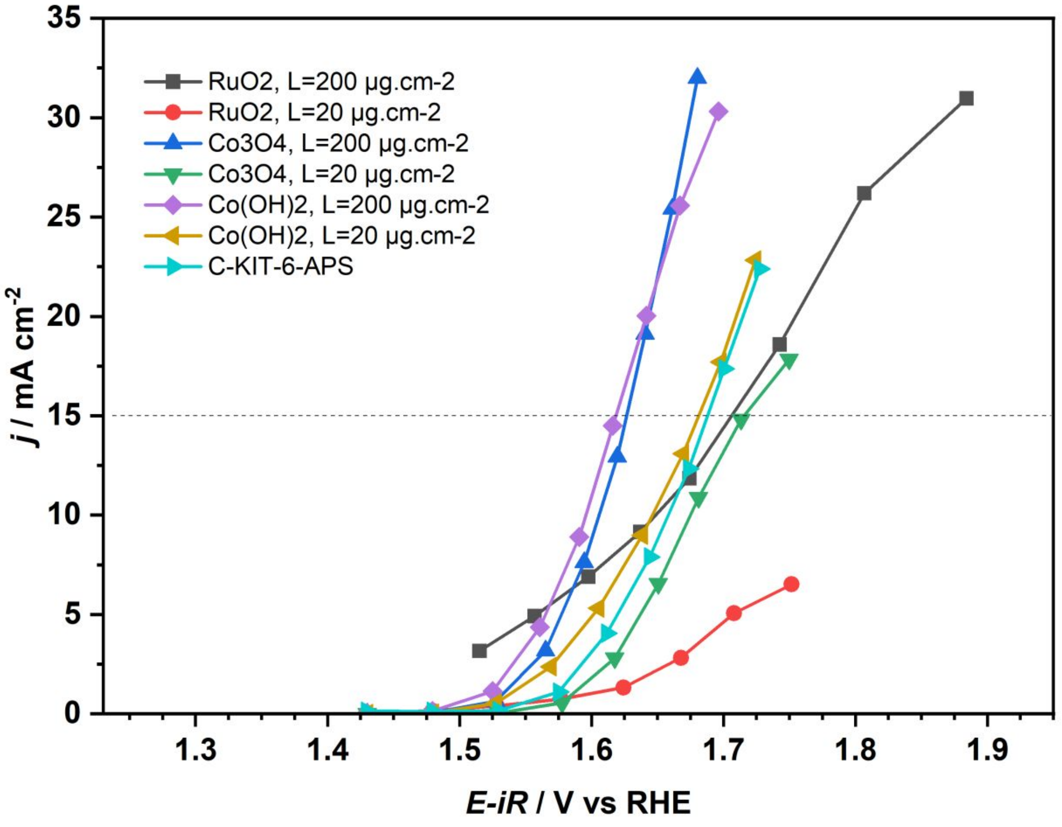

Figure S 5 Comparison of OER activity pf C_{KIT-6}-APS-Co with reference materials at different loadings.

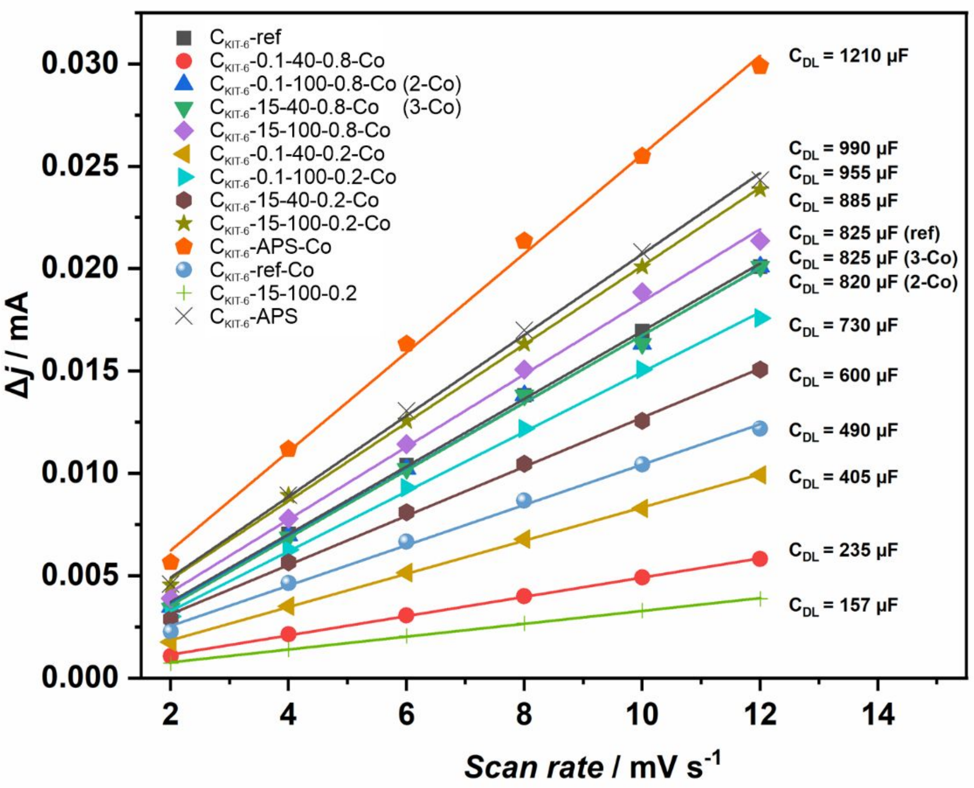

Figure S 6 Data points for C_{DL} calculation for cobalt-doped C_{KIT-6} samples.

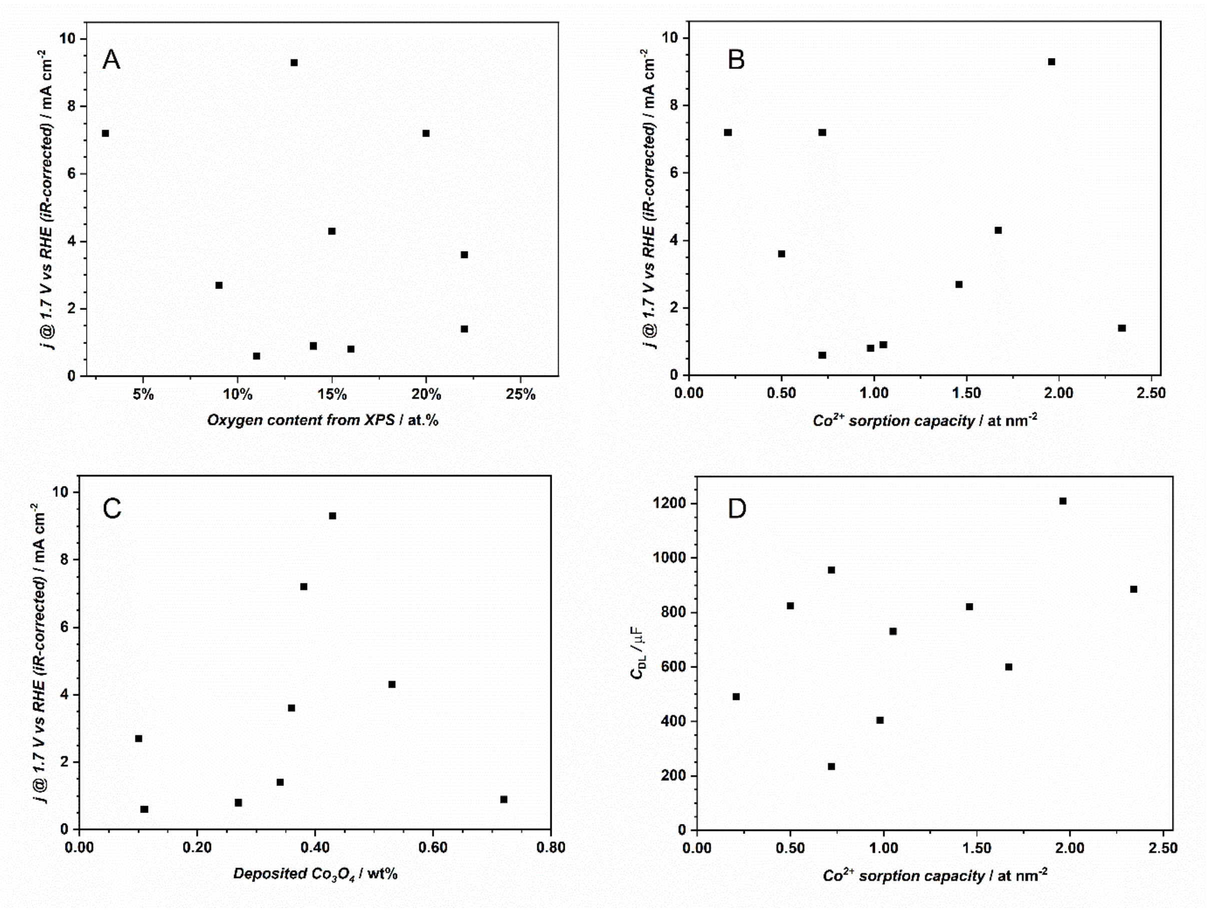

Figure S 7 Analysis of possible correlations between surface properties and electrochemical properties of modified C_{KIT-6}.

Table S 2 Relative content of oxygen functional groups on modified C_{KIT-6} carbons. C1 are C–O-type; C2 are C=O-type; C3 are COO-type groups.

Sample name	C1/(C1 + C2 + C3)	C2/(C1 + C2 + C3)	C3/(C1 + C2 + C3)
C_{KIT-6}-ref	1.00	0.00	0.00
C_{KIT-6}-0.1-40-0.8	0.55	0.32	0.13
C_{KIT-6}-0.1-100-0.8	0.62	0.19	0.19
C_{KIT-6}-15-40-0.8	0.24	0.47	0.29
C_{KIT-6}-15-100-0.8	0.24	0.45	0.31
C_{KIT-6}-0.1-40-0.2	0.20	0.53	0.27
C_{KIT-6}-0.1-100-0.2	0.28	0.47	0.25
C_{KIT-6}-15-40-0.2	0.28	0.37	0.34
C_{KIT-6}-15-100-0.2	0.33	0.31	0.37
C_{KIT-6}-APS	0.43	0.14	0.43

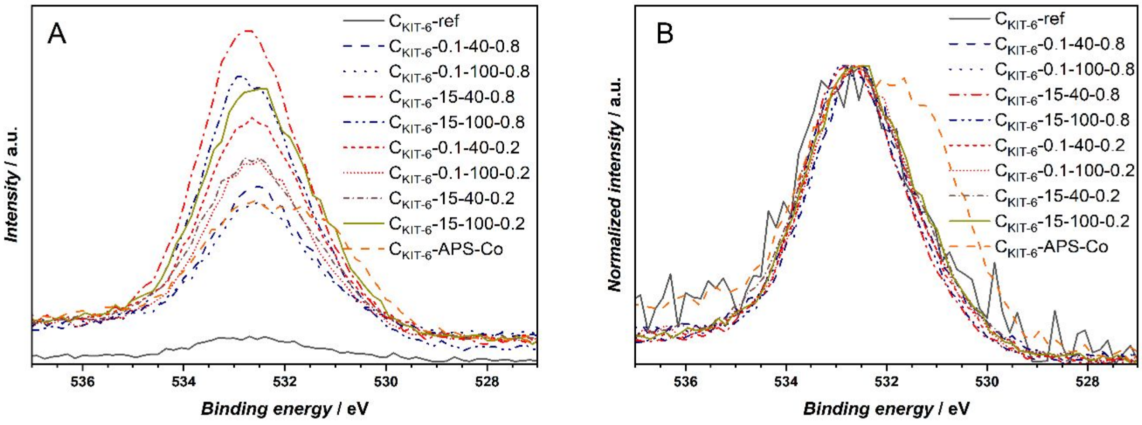

Figure S 8 XPS spectra in the O 1s range of C_{KIT-6} samples. A) Comparison of intensities, B) comparison of peak positions.

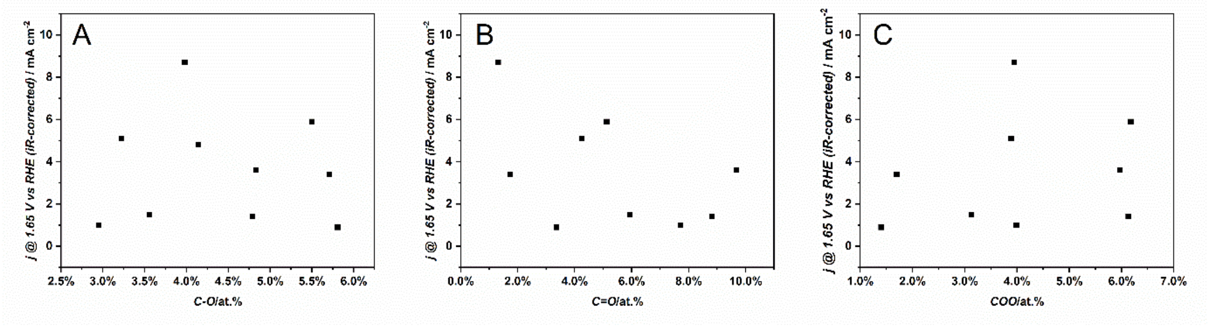

Figure S 9 Analysis of possible correlations between the atomic concentration of surface oxygen species and OER performance of modified C_{KIT-6}.

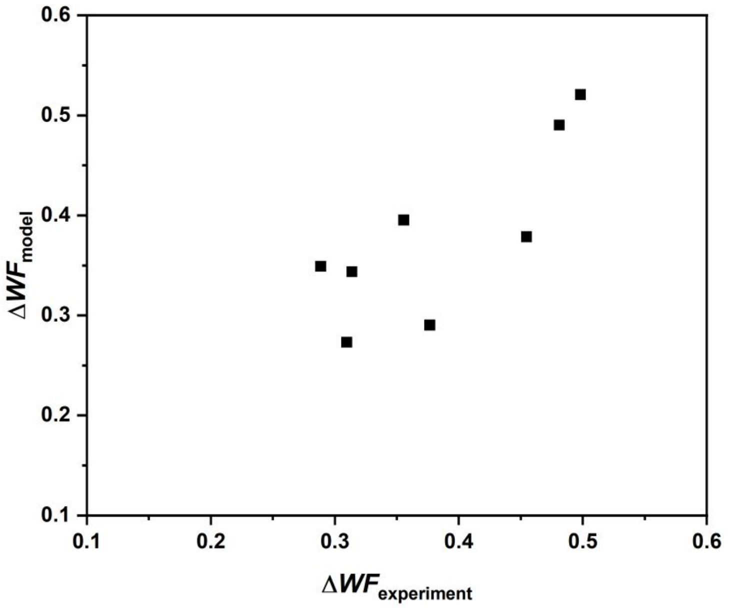

Figure S 10 Results of linear regression analysis of contribution to work function changes of different oxygen surface groups (C1, C2, C3).

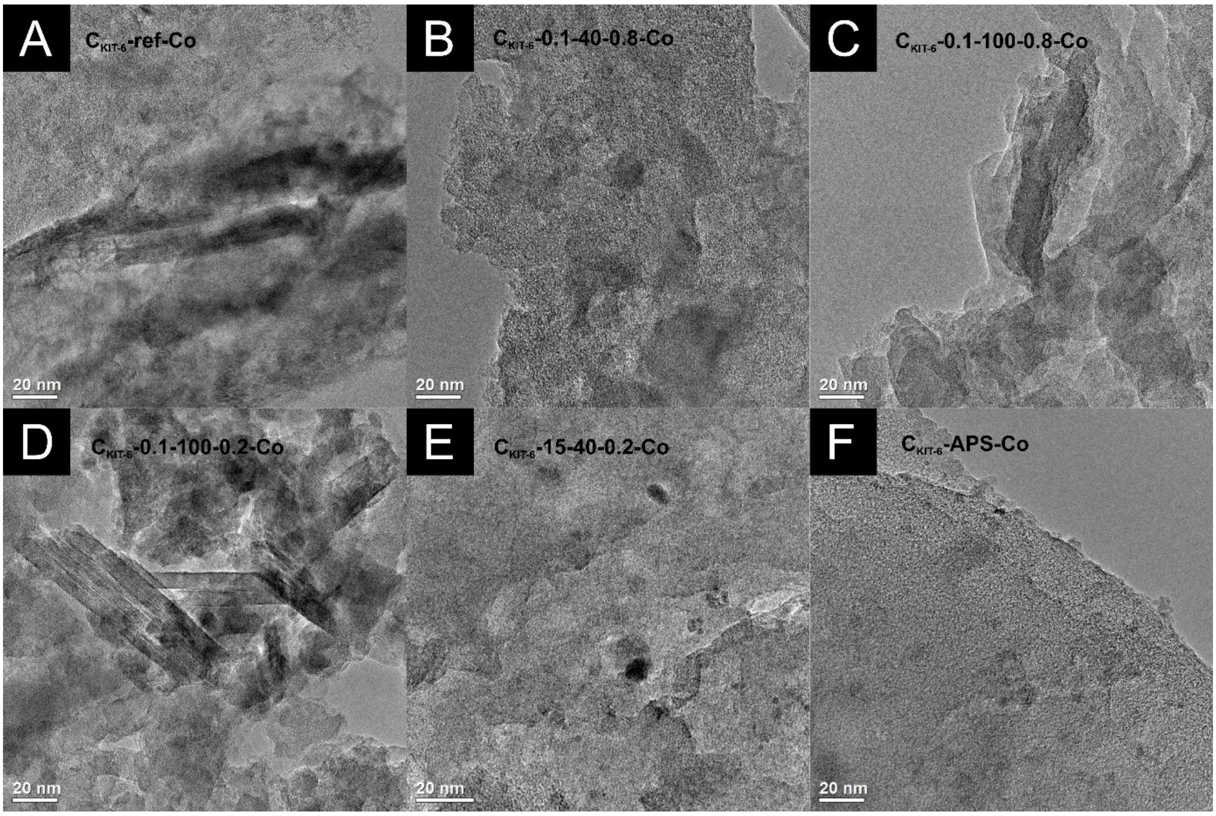

Figure S 11 TEM pictures of A) C_{KIT-6}-ref-Co, B) C_{KIT-6}-0.1-40-0.8-Co, C) C_{KIT-6}-0.1-100-0.8-Co, D) C_{KIT-6}-0.1-100-0.2-Co, E) C_{KIT-6}-15-40-0.2-Co, and F) C_{KIT-6}-APS-Co.

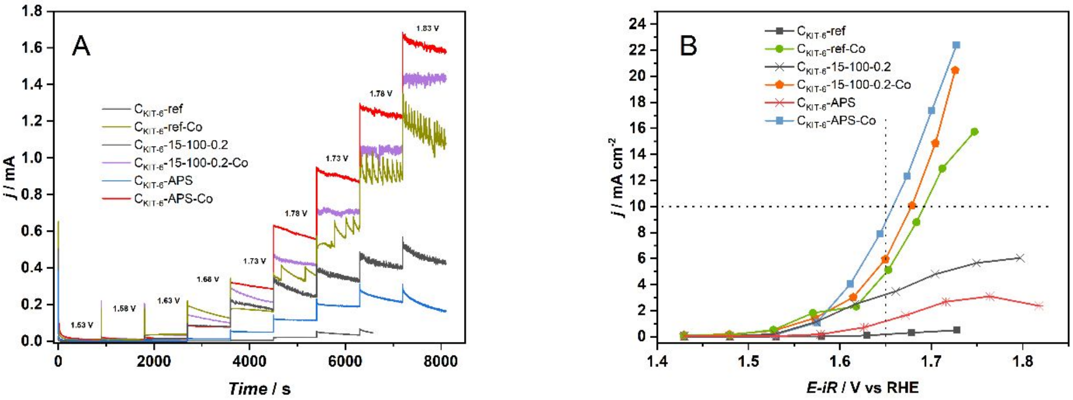

Figure S 12 A) Chronoamperometric results of oxygen evolution reaction studies on oxidized C_{KIT-6} materials, B) Comparison of OER activity of plasma and APS-oxidized C_{KIT-6} with cobalt-promoted C_{KIT-6}.

Table S 3 Acid-base properties of reference, APS-modified and plasma-modified C_{KIT-6} samples.

**Sample**	**Acidic groups (mmol g^{-1})**	**Basic groups (mmol g^{-1})**	**Total groups (mmol g^{-1})**
C_{KIT-6}-ref	2.46	0.25	2.71
C_{KIT-6} 10 min O_2 plasma	1.74	0.75	2.49
C_{KIT-6}-APS	4.03	0.00	4.03
